# Multi-modular metabolic engineering and efflux engineering for enhanced lycopene production in recombinant *Saccharomyces cerevisiae*

**DOI:** 10.1093/jimb/kuae015

**Published:** 2024-04-15

**Authors:** Guangxi Huang, Jiarong Li, Jingyuan Lin, Changqing Duan, Guoliang Yan

**Affiliations:** C entre for Viticulture and Enology, College of Food Science and Nutritional Engineering, China Agricultural University, Beijing 100083, China; Key Laboratory of Viticulture and Enology, Ministry of Agriculture and Rural Affairs, Beijing 100083, China; C entre for Viticulture and Enology, College of Food Science and Nutritional Engineering, China Agricultural University, Beijing 100083, China; Key Laboratory of Viticulture and Enology, Ministry of Agriculture and Rural Affairs, Beijing 100083, China; C entre for Viticulture and Enology, College of Food Science and Nutritional Engineering, China Agricultural University, Beijing 100083, China; Key Laboratory of Viticulture and Enology, Ministry of Agriculture and Rural Affairs, Beijing 100083, China; C entre for Viticulture and Enology, College of Food Science and Nutritional Engineering, China Agricultural University, Beijing 100083, China; Key Laboratory of Viticulture and Enology, Ministry of Agriculture and Rural Affairs, Beijing 100083, China; C entre for Viticulture and Enology, College of Food Science and Nutritional Engineering, China Agricultural University, Beijing 100083, China; Key Laboratory of Viticulture and Enology, Ministry of Agriculture and Rural Affairs, Beijing 100083, China; Key Laboratory of Food Bioengineering (China National Light Industry), China Agricultural University, Beijing 100083, China

**Keywords:** Lycopene, *Saccharomyces cerevisiae*, Multi-modular, Acetate, Efflux

## Abstract

Lycopene has been widely used in the food industry and medical field due to its antioxidant, anti-cancer, and anti-inflammatory properties. However, achieving efficient manufacture of lycopene using chassis cells on an industrial scale remains a major challenge. Herein, we attempted to integrate multiple metabolic engineering strategies to establish an efficient and balanced lycopene biosynthetic system in *Saccharomyces cerevisiae*. First, the lycopene synthesis pathway was modularized to sequentially enhance the metabolic flux of the mevalonate pathway, the acetyl-CoA supply module, and lycopene exogenous enzymatic module. The modular operation enabled the efficient conversion of acetyl-CoA to downstream pathway of lycopene synthesis, resulting in a 3.1-fold increase of lycopene yield. Second, we introduced acetate as an exogenous carbon source and utilized an acetate-repressible promoter to replace the natural *ERG9* promoter. This approach not only enhanced the supply of acetyl-CoA but also concurrently diminished the flux toward the competitive ergosterol pathway. As a result, a further 42.3% increase in lycopene production was observed. Third, we optimized NADPH supply and mitigated cytotoxicity by overexpressing ABC transporters to promote lycopene efflux. The obtained strain YLY-PDR11 showed a 12.7-fold increase in extracellular lycopene level compared to the control strain. Finally, the total lycopene yield reached 343.7 mg/L, which was 4.3 times higher than that of the initial strain YLY-04. Our results demonstrate that combining multi-modular metabolic engineering with efflux engineering is an effective approach to improve the production of lycopene. This strategy can also be applied to the overproduction of other desirable isoprenoid compounds with similar synthesis and storage patterns in *S. cerevisiae*.

**One-Sentence Summary:**

In this research, lycopene production in yeast was markedly enhanced by integrating a multi-modular approach, acetate signaling-based down-regulation of competitive pathways, and an efflux optimization strategy.

## Graphical Abstract

**Graphical Abstract:**
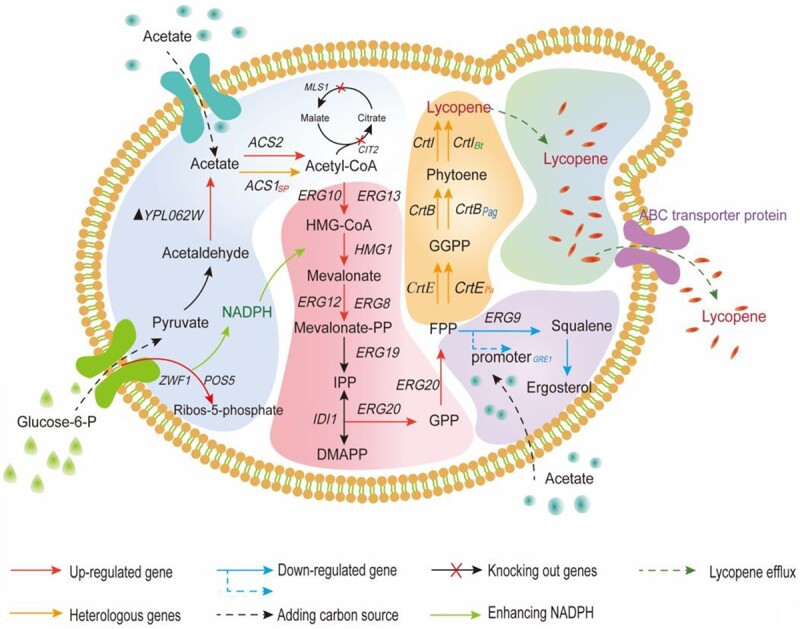

## Introduction

Lycopene (C40H56), a quintessential carotenoid, is extensively utilized in pharmaceutical formulations, dietary supplements, functional foods, and cosmetic additives, owing to its antioxidative, anticarcinogenic, and anti-inflammatory properties (Jing et al., 2021; Kapała et al., 2022). At present, natural carotenoids are mainly extracted from plants, algae, and microorganisms, which is far from satisfying the needs of consumers (Jing et al., 2022). With the rapid development of synthetic biology, some model microorganisms have been genetically engineered for carotenoid production, such as *Saccharomyces cerevisiae*, a promising platform for lycopene synthesis with safety, robustness, and genetic tractability characteristics (Paramasivan & Mutturi, 2017).

**Fig. 1.**
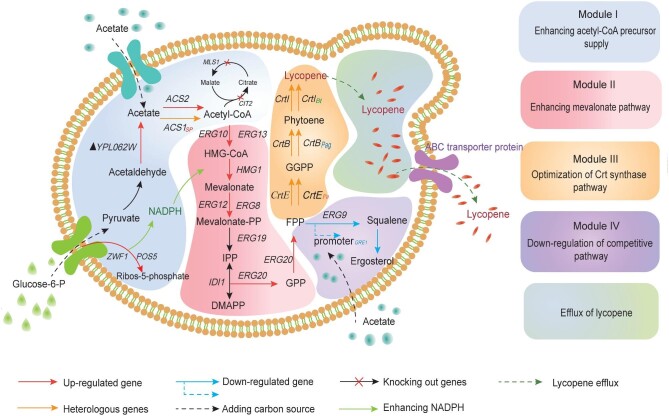
Schematic of the multi-modular engineering and efflux strategies for lycopene synthesis in *Saccharomyces cerevisiae*. Module I (Acetyl-CoA Supply) supplies the essential precursor acetyl-CoA for lycopene production. Module II (MVA Pathway) involves the overexpression of key enzymes to redirect carbon flux toward the triterpenoid precursor. Module III (lycopene Synthesis) incorporates exogenous genes for lycopene synthesis in *S. cerevisiae*. Module IV (Competitive Pathway) involves the downregulation of the ergosterol synthesis pathway via acetate. Intracellular lycopene is secreted from the cell by the overexpression of an ABC transporter. *ZWF1*, glucose-6-phosphate dehydrogenase; *ACS1*, acetate-CoA ligase; *ACS2*, acetate-CoA ligase; *MLS1*, malate synthase; *CIT2*, citrate synthase; *ERG10*, acetyl-CoA acetyltransferase; *ERG13*, hydroxymethylglutaryl-CoA synthase; *tHMG1*, truncated HMG-CoA reductase; *ERG12*, mevalonate kinase; *ERG8*, phosphomevalonate kinase; *ERG19*, diphosphomevalonate decarboxylase; *ERG20*, farnesyl pyrophosphate synthetase; *IDI1*, isopentenyl diphosphate isomerase; *ERG9*, squalene synthase; IPP, isopentenyl pyrophosphate; DMAPP, dimethylallyl pyrophosphate; and FPP, farnesyl.

The lycopene biosynthesis pathway has been extensively studied and well characterized in *S. cerevisiae* (Zhang et al., 2021), and can be divided into three distinct metabolic modules (Fig. 1). Module I, the central carbon pathway, features acetyl-CoA as a crucial metabolic node. Acetyl-CoA is a key precursor of the upstream pathway for lycopene synthesis, which affects cell growth and a variety of metabolic pathways (Krivoruchko et al., 2015). Module I also provides the cofactors nicotinamide adenine dinucleotide phosphate (NADPH) and ATP required for the downstream pathway (Ma et al., 2019). Module II involves the mevalonate (MVA) pathway, which generates the precursors isopentenyl pyrophosphate and dimethylallyl pyrophosphate from acetyl-CoA through a multi-step enzymatic reactions (Wang et al., 2016; Zhang et al., 2021). These two precursors are catalyzed by pyrophosphate synthetase (FPPS/ERG20) to form farnesyl pyrophosphate (FPP) (Ignea et al., 2015). Module Ⅲ requires the introduction of an exogenous lycopene synthetic pathway. Subsequently, FPP is catalyzed by geranylgeranyl diphosphate synthase (GGPPS/CrtE) to yield the lycopene precursor geranylgeranyl diphosphate (GGPP). Then, two GGPP molecules are condensed to phytoene by phytoene synthase (CrtB), and lycopene is synthesized by four-step dehydrogenation under the action of exogenous phytoene desaturase (CrtI) (Li et al., 2020; Luo et al., 2020). In addition, the competitive ergosterol pathway can be divided into module IV, which is the main competitive pathway for *S. cerevisiae* to consume FPP (Peng et al., 2017). By reducing or inhibiting the ergosterol pathway, it's possible to minimize the loss of carbon flux and enhance the metabolic flow directed toward target products, thus effectively boosting the synthesis of carotenoids (Ma et al., 2019).

To achieve higher lycopene production, the carotenoid pathway needs to acquire more metabolic flux. The refined modularization makes it more convenient to assess and strengthen the flux into the carotenoid synthesis pathway (Jin et al., 2019). For each metabolic module, the limiting factors for metabolic flow are different, thus it is necessary to apply specialized modification strategies. In Module I, insufficient supply of acetyl-CoA is the main factor limiting carotenoid production. The strategies such as overexpression of acetyl-CoA synthetase (Liu et al., 2013), reduction of acetyl-CoA consumption (Lian et al., 2014), and alternative consumption of acetyl-CoA in the peroxisome are usually used to alleviate the shortage of its supply (Su et al., 2022). In Module II, flux enhancement of the MVA pathway is restrained by several rate-limiting steps due to the complexity of MVA pathway regulation. The effective strategies include increasing key gene copy number (Huo et al., 2019), promoter engineering (Deaner & Alper, 2018), ribosome binding site engineering (Shi et al., 2016), and directed evolution of key genes in MVA pathway (Xie et al., 2015). For example, the low expression level of HMG-CoA reductase is the major rate-limiting step in the MVA pathway. Overexpression of truncated *Hmg1* (*tHmg1*) can increase the supply of terpenoid precursors in engineered yeast (Ohto et al., 2009). Additionally, the unique intracellular microenvironment of *S. cerevisiae* can result in heterologous terpenoid synthases experiencing low expression levels and suboptimal enzyme activity (Chang et al., 2015). Thus, screening of enzymes from different sources for Module Ⅲ and subsequent modification is a powerful way to improve targeted product synthesis (Xie et al., 2015). In Module IV, the squalene synthetase gene (*ERG9*) is the first gene in the ergosterol biosynthetic pathway. Dynamically down-regulating *ERG9* expression by replacing the original promoter with a specific promoter can redirect the metabolic flux toward the carotenoid pathway. Examples include *P*_MET3_, inhibited by methionine (Asadollahi et al., 2008); *P*_HXT1_, induced by glucose (Özaydın et al., 2013); *P*_CTR3_, inhibited by copper (Paddon et al., 2013); and *P*_IZH1_, inhibited by oleic acid (Bu et al., 2022). Continuing to explore repressible promoters for *ERG9* expression, which can adapt to variations in nutrients and other environmental fluctuations, remains a challenge in further enhancing carotenoid production.

As a hydrophobic substance, lycopene, due to its large accumulation in cells, can cause cytotoxicity and cell growth inhibition (Zhu et al., 2015). Efflux engineering is a highly promising approach to mitigate the toxicity of products and improve cell growth (Hara et al., 2017). *Saccharomyces cerevisiae* contains 11 plasma membrane ATP-binding cassette (ABC) transporters, enabling cells to efficiently pump a myriad of substances and withstand harsh conditions and toxic compounds (Prasad & Goffeau, 2012). Our previous study indicated that overexpression of the ABC transporter Snq2p can significantly enhance β-carotene secretion and intracellular production in yeast (Bu et al., 2020). Therefore, mining specific endogenous ABC transporters for lycopene efflux is another promising approach to promote its production. However, there are no reports systematically evaluating the effect of these 11 ABC endogenous transporters on lycopene efflux in *S. cerevisiae.*

In this study, aimed at enhancing the lycopene yield of *S. cerevisiae*, a comprehensive array of metabolic engineering strategies was used to modify various lycopene synthesis modules. First, we overexpressed various sources of *ACS1* to promote acetyl-CoA accumulation, combined with upregulating key genes of the MVA pathway and introducing additional lycopene synthesis genes to promote the flux to lycopene synthetic module. Second, based on previous transcriptomic data, we replaced the native *ERG9* promoter with an acetate-repressible *GRE1* promoter, dynamically reducing *ERG9* expression. This shift redirected metabolic flux toward the lycopene pathway under acetate stress, resulting in a 42.3% increase in lycopene content. Finally, after enhancing the supply of NADPH, we explored the capacity of 11 ABC transporters efflux lycopene and identified PDR11 as the most efficient transporter. A 12.7-fold increase in lycopene secretion and a 6.9% increase in intracellular lycopene production were achieved in the final engineered strain compared with the reference strain. This work demonstrates the combination of multiple module strategies and efflux engineering is an effective way to improve the synthesis of carotenoid in *S. cerevisiae* cell factory.

## Materials and Methods

### Strains, Media, and Reagents

*Escherichia coli* strain DH5α was used for plasmid construction and amplification. All *S. cerevisiae* strains used in this study are listed in Table 1. The strain YLY-01 was used to establish a heterologous lycopene pathway by integrating genes encoding GGPP synthase (CrtE), phytoene synthase (CrtB), and phytoene desaturase (CrtI) from *Xanthophyllomyces dendrorhous* (Li et al., 2023). These genes were placed under the control of the galactose-regulated *GAL* promoter (Xie et al., 2014). Luria–Bertani medium (10 g/L tryptone, 5 g/L yeast extract, and 10 g/L NaCl) was used for *E. coli* cultivation. Kanamycin (50 mg/mL) was used for selections. Yeast extract-peptone-dextrose (YPD) medium (10 g/L yeast extract, 20 g/L peptone, and 20 g/L glucose) was used for the cultivation of yeast strains for competent cell preparation and shake flask fermentation. Yeast extract-peptone-dextrose medium supplemented with 200 μg/mL geneticin was used for KanMX marker selection. SD-FOA (SD complete medium with 1 mg/mL 5-fluoroorotic acid) was utilized to select yeast strains with KanMX-URA3-PRB322ori marker excision.

**Table 1.** *Saccharomyces cerevisiae* Strains Used in This Study

Strain	Host strain	Genotype/description	Source
FY1679-01B	S288C	*MATa; ura3-52*	EUROSCARF
YLY-01B	FY1679-01B	*ΔGAL1-10-7::T_ADH1_-CrtI-P_GAL10_-P_GAL1_-CrtB-T_CYC1_*	This lab
YLY-01	YLY-01B	Δ*GAL8*0:: *T_ADH1_*-*CrtE*-*P_GAL10_*	This lab
YLY-04	YLY-01	Δ*YPL062W*:: *T_ADH1_-ACS2-P_GAL10_*	This lab
YLY-21	YLY-04	Δ*MLS1*	This study
YLY-22	YLY-04	Δ*MLS1*:: *P_GAL1_-ACS1_SP_-T_CYC1_*	This study
YLY-23	YLY-04	Δ*MLS1*:: *P_GAL1_- ACS1_YL_-T_CYC1_*	This study
YLY-24	YLY-04	Δ*MLS1*:: *P_GAL1_-ACS1_SE_ ^L641P^-T_CYC1_*	This study
YLY-25	YLY-04	Δ*CIT2*	This study
YLY-26	YLY-04	Δ*CIT2* :: *T_ADH1_-ERG10-P_GAL10_*	This study
YLY-27	YLY-04	Δ*CIT2* :: *T_ADH1_-ERG12-P_GAL10_*	This study
YLY-28	YLY-04	Δ*CIT2* :: *T_ADH1_-ERG13-P_GAL10_*	This study
YLY-29	YLY-04	Δ*CIT2* :: *T_ADH1_-ERG13-P_GAL10_*	This study
YLY-30	YLY-04	Δ*CIT2* :: *T_ADH1_-tHMG1-P_GAL10_*	This study
YLY-31	YLY-26	Δ*ROX1*	This study
YLY-32	YLY-31	Δ*ROX1*:: *T_ADH1_-ERG12-P_GAL10_*	This study
YLY-33	YLY-31	Δ*ROX1*::*T_ADH1_-ERG12-P_GAL10_-P_GAL1_-ERG20-T_CYC1_*	This study
YLY-34	YLY-33	Δ*DOS2*	This study
YLY-35	YLY-33	Δ*DOS2*:: *T_ADH1_-tHMG1-P_GAL10_*	This study
YLY-36	YLY-35	Δ*MLS1*	This study
YLY-37	YLY-35	Δ*MLS1*:: *P_GAL1_-ACS1_SP_-T_CYC1_*	This study
YLY-38	YLY-35	Δ*MLS1*:: *P_GAL1_-ACS1_YL_-T_CYC1_*	This study
YLY-39	YLY-35	Δ*MLS1*:: *P_GAL1_-ACS1_SE_ ^L641P^-T_CYC1_*	This study
YLY-40	YLY-35	Δ*HO*:: *T_ADH1_-CrtI-P_GAL10_*	This study
YLY-41	YLY-40	Δ*PAH1*:: *T_ADH1_-CrtB-P_GAL10_-P_GAL1_-CrtE-T_CYC1_*	This study
YLY-42	YLY-37	Δ*HO*:: *T_ADH1_-CrtI-P_GAL10_*	This study
YLY-43	YLY-42	Δ*PAH1*:: *T_ADH1_-CrtB-P_GAL10_-P_GAL1_-CrtE-T_CYC1_*	This study
YLY-44	YLY-43	Δ*LPP1*:: *P_GAL1_-SET5-T_CYC1_*	This study
YLY-45	YLY-44	Δ*DPP1*:: *T_ADH1_-HST4-P_GAL10_*	This study
YLY-46	YLY-45	Δ*P_ERG9_*::*P_HOR7_*	This study
YLY-47	YLY-45	Δ*P_ERG9_*::*P_GRE1_*	This study
YLY-48	YLY-45	Δ*P_ERG9_*::*P_SPS100_*	This study
YLY-49	YLY-45	Δ*P_ERG9_*::*P_DPA10_*	This study
YLY-50	YLY-47	Δ*TY4*:: *PGAL1-POS5-T_CYC1_*	This study
YLY-51	YLY-47	Δ*TY4*:: *PGAL1-ZWF1-T_CYC1_*	This study
YLY-PDR5	YLY-50	Δ*P_PDR5_*::*P_GAL1_*	This study
YLY-PDR10	YLY-50	Δ*P_PDR10_*::*P_GAL1_*	This study
YLY-PDR11	YLY-50	Δ*P_PDR11_*::*P_GAL1_*	This study
YLY-PDR12	YLY-50	Δ*P_PDR12_*::*P_GAL1_*	This study
YLY-PDR15	YLY-50	Δ*P_PDR15_*::*P_GAL1_*	This study
YLY-PDR18	YLY-50	Δ*P_PDR18_*::*P_GAL1_*	This study
YLY-STE6	YLY-50	Δ*P_STE6_*::*P_GAL1_*	This study
YLY-SNQ2	YLY-50	Δ*P_SNQ2_*::*P_GAL1_*	This study
YLY-YOR1	YLY-50	Δ*P_YOR1_*::*P_GAL1_*	This study
YLY-YOL075C	YLY-50	Δ*P_YOL075C_*::*P_GAL1_*	This study
YLY-AUS1	YLY-50	Δ*P_AUS1_*::*P_GAL1_*	This study

The DH5α strain of *E. coli*, the plasmid extraction kit, and lysozyme were acquired from Tiangen Biochemical Technology (Beijing, China). Standardized lycopene was obtained from Yuanye Biotech (Shanghai, China), while sodium acetate was sourced from Macklin Biochemical (Shanghai, China). All other chemicals were supplied by Sigma–Aldrich (USA).

### DNA Manipulation, Plasmid Construction, and Strain Construction

All plasmids and primers are detailed in Supplementary Tables S1 and S2, respectively. All primers were ordered from Sangon Biotech (Shanghai, China). Gene codons were optimized using the IDT website (https://www.idtdna.com/), then the sequences were synthesized by Synbio Technologies (Suzhou, China) (Supplementary Table S3). The plasmids were ligated using the One Step Cloning Kit purchased from Vazyme Biotech (Nanjing, China), which facilitates ligation of two or more DNA fragments by overlapping 20 bp. From the genomic DNA of *S. cerevisiae* FY1679-01B, DNA fragments, promoters, and homologous arms were amplified by PCR. As previously reported, the pUMRI constructs were linearized from the homologous arm junctions and integrated into the yeast genome by lithium acetate/polyethylene glycol/single-stranded carrier DNA transformation (Gietz & Schiestl, 2007). Genomic DNA PCR verification primers facilitated the evaluation of all transformants (Supplementary Fig. S1). Following PCR confirmation, the correct colonies were grown at 30°C, 220 rpm for 12 hr. Due to URA3 excision, further recombination between the duplicated Loxp flanks results in 5-FOA resistance. 5-FOA-resistant colonies were picked and screened for target marker loss by replicating on YPD and YPD (G418) plates. Detailed construction procedure of the promoter replacement plasmids is shown in (Supplementary Fig. S2).

### Fermentation Conditions

Single colonies were selected from the plate and inoculated into 4 mL YPD liquid medium for overnight growth at 30°C, 220 rpm in dark conditions. The seed culture was then used to inoculate 250 mL flasks containing 50 mL of YPD medium to an initial optical density (OD_600_) of 0.05 and grown under the same conditions for 72 hr. Cell growth was monitored by measuring OD_600_ on a spectrophotometer. N-dodecane was selected as an organic phase because of its potent hydrophobicity and low toxicity to saccharomyces cerevisiae cells (Ling et al., 2013). During two-phase fermentation, N-dodecane was incorporated into the medium at a 1:10 volume ratio was filtered through a 0.22 μm organic needle strainer before addition to the flask.

### Lycopene Extraction and Quantification

Lycopene was extracted intracellularly using hot HCl acetone (Xie et al., 2014). The analyses of lycopene were performed on a High-Performance Liquid Chromatography (HPLC) system (Agilent 1200 LC) equipped with a C18 column (5 μm, 4.6 mm × 150 mm) and the UV/VIS signals were detected at 472 nm. The mobile phase was acetonitrile-methanol–isopropanol (50:30:20 v/v). The flow rate was 1 mL/min at 40°C. The standard curve was prepared for the quantification of lycopene (Supplementary Fig. S3a). The lycopene in the dodecane layer was determined by ultraviolet spectrophotometer at 472 nm. A standard curve was also prepared for lycopene quantification (Supplementary Fig. S3b). The production of lycopene was expressed as grams per liter of fermentation broth (g/L) and milligrams per gram of dry cell weight (mg/g DCW). To determine the DCW, cells from 1 mL of culture were collected by centrifugation at 12 000 × g for 1 min and rinsed twice with distilled water. The cells were then dried at 80°C until a constant weight was reached and weighed to calculate DCW.

### Quantification of Acetyl-CoA

Cells were sampled during the course of lycopene shake-flask fermentation (mid-log phase) for the acetyl-CoA assay. The harvested cells were washed twice with precooled PBS and then mixed thoroughly with 600 μL sorbitol buffer and 50 U lysozyme. The cells were treated at 30°C for 30 min, followed by centrifugation at 4000 × g for 10 min to remove the precipitate. The supernatant was then treated with RIPA cracking solution (strong). Acetyl-CoA concentration was determined using an enzyme-linked immunoassay kit purchased from Jingmei Biotech (Jiangsu, China). The acetyl-CoA concentration was averaged from biological duplicates and normalized to DCW.

### Quantitative Real‑Time PCR (qRT‑PCR) Analysis

The HiPure Yeast RNA Kit (Magen, Guangzhou, China) was used to isolate total RNA from harvested yeast cells. The HiScript® II Q RT SuperMix for qPCR (gDNA wiper) (Vazyme, Nanjing, China) was used to reverse transcribe the treated total RNA. Specific primers were designed (Supplementary Table S2) and used in qRT-PCR for gene expression analysis. To normalize the different samples, the housekeeping gene *ACT1* was used as a reference gene. The 2^−ΔΔCT^ method was used for relative gene expression analysis (Livak & Schmittgen, 2001).

### Statistical Analysis

All experiments were repeated three times. The standard error was estimated through the square root of the estimated error variance of the quantity. Statistical significance (*p* < 0.05) was determined by Student's *t*-tests.

## Results

### Enhancing the Supply of Acetyl-CoA Precursor By Introducing Different Exogenous *ACS1*

**Fig. 2.**
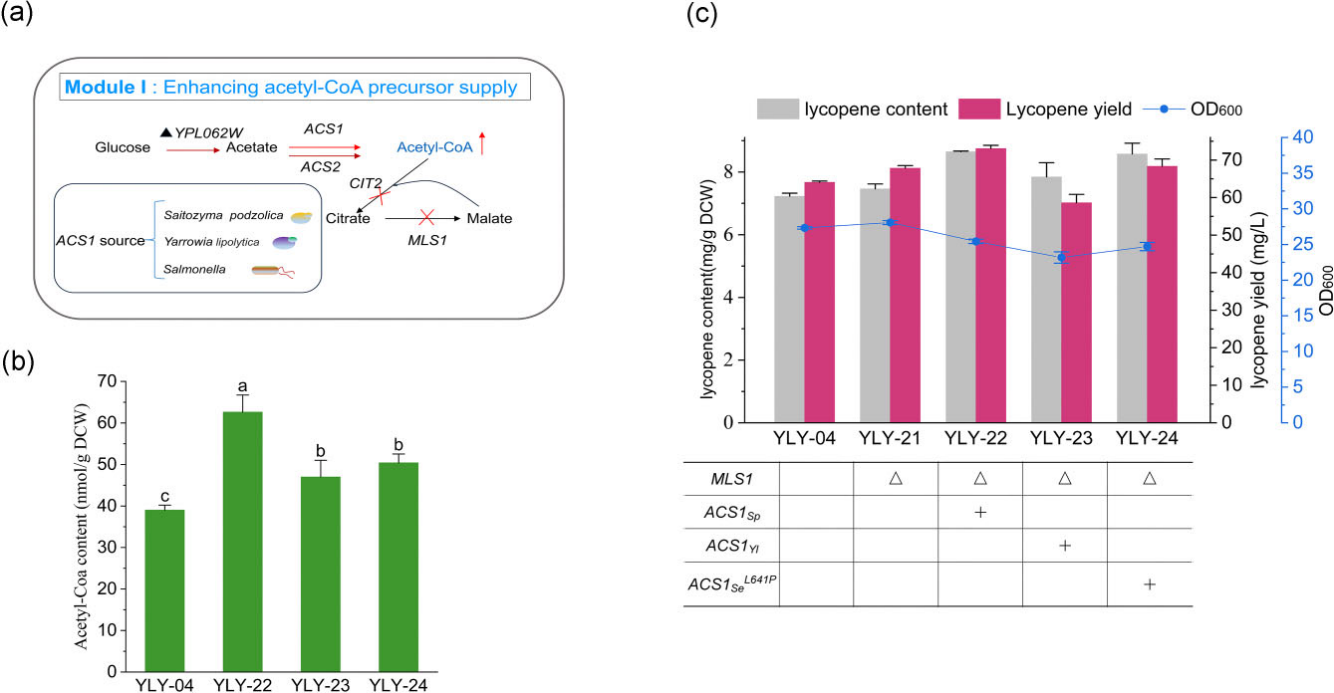
Enhancing the Acetyl-CoA pool by introducing various sources of *ACS1*. (a) Overview of genetic modifications of Module I, (b) Acetyl-CoA content, and (c) the cell growth, lycopene content, and yield of recombinant strains. Note: Different lowercase letters indicated significant differences among different groups: *p *< 0.05. Data are the means ± standard deviations of triplicate experiments.

In this study, YLY-04 serves as the initial host for subsequent modification (Li et al., 2023). After 72 hr of fermentation, the lycopene content and yield of strain YLY-04 reached 7.4 mg/g DCW and 64.3 mg/L, respectively. To ensure sufficient precursor acetyl-CoA, we initially modified module I by overexpressing various sources of *ACS1* in strain YLY-04 (Fig. 2a). Deletion of *MLS1* can promote the increase of acetyl-CoA (Krivoruchko et al., 2013). Thus, we knocked out *MLS1* as homologous arms to obtain the strain YLY-21, however, the lycopene content only increased by 3.3% compared with that of the control YLY-04. On this basis, we integrated three different acetyl-CoA synthesis gene *ACS1* into YLY-21 including *ACS1_Sp_* (*Saitozyma podzolica*), *ACS1_Yl_* (*Yarrowia lipolytica*), *ACS1_Se_  ^L641P^* (*Salmonella*), and constructed three strains YLY-22, YLY-23, and YLY-24, respectively. Following genetic modification, the intracellular acetyl-CoA levels of all recombinant strains significantly increased (Fig. 2b), among which YLY-22 (*ACS1_Sp_*) showed the highest accumulation with 60.5% increase compared to the control YLY-21, reaching 62.7 nmol/g DCW. However, the observed increase in intracellular acetyl-CoA did not correspondingly result in a significant increase in lycopene synthesis, with only a 16.6% increment observed in strain YLY-22, yielding 8.7 mg/g DCW of lycopene (Fig. 2c). Notably, the growth of all recombinant strains was inhibited to different extents (Fig. 2c). Given the negative effect of acetyl-CoA accumulation in the cell and the low promotion of lycopene synthesis, we speculated that the conversion of acetyl-CoA might be blocked by the downstream MVA pathway, as the MVA pathway is subject to complex transcriptional regulation (Lv et al., 2014). Therefore, before increasing the synthesis of acetyl-CoA, we attempted to prioritize enhancing the metabolic flux of the MVA pathway.

### Optimizing MVA Pathway Flux for Enhanced Lycopene Production

**Fig. 3.**
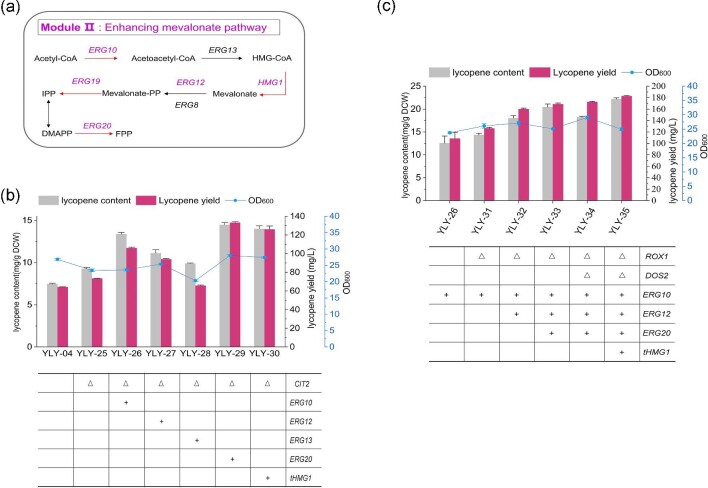
Overexpressing MVA pathway genes to promote acetyl-CoA metabolism. (a) Overview of genetic modifications of Module Ⅱ, (b) the cell growth, lycopene content, and yield in recombinant strains with singular overexpression of key genes in the MVA pathway, and (c) the cell growth, lycopene content, and yield of recombinant strains with multiple overexpression of key genes in the MVA pathway. Data are the means ± standard deviations of triplicate experiments.

To evaluate the effect of engineering module Ⅱ (MVA pathway) on subsequent lycopene synthesis, we selected five key genes of the MVA pathway, including *ERG10, ERG12, ERG13, ERG20*, and *tHMG1*, for single and combined overexpression (Fig. 3a). Here, the recombinant strain YLY-04 remained as the initial host, and *CIT2* was knocked out as the homologous arm. The five genes were firstly individually overexpressed with the *GAL* promoter to obtain the recombinant yeast YLY-26 to YLY30, respectively (Fig. 3b). Overexpression of *ERG10, ERG12, ERG20*, and *tHMG1* further increased lycopene yield, whereas overexpression of *ERG13* in strain YLY-28 resulted in decreased lycopene yield due to strong inhibition of cell growth. The highest increase was observed in the strain YLY-29 (*ERG20*), followed by YLY-30 (*tHMG1*), YLY-26 (*ERG10*), and YLY-27 (*ERG12*). Subsequently, we examined the effect of simultaneously overexpressing multiple genes in the MVA pathway on lycopene synthesis. Based on strain YLY-26(*ERG10*), we used *ROX1* and *DOS2* as homologous arms to overexpress *ERG12, ERG20*, and *tHMG1* one by one to construct strains YLY-32 (*ERG10 + ERG12*), YLY-33 (*ERG10 + ERG12 + ERG20*), and YLY-35 (*ERG10 + ERG12 + ERG20 + tHMG1*). As expected, lycopene production was gradually enhanced with the increased number of overexpressed genes (Fig. 3c). The highest lycopene content (20.8 mg/g DCW) was thus produced in the strain YLY-35 with four overexpressed genes, which was 1.8-fold higher than that of the initial strain YLY-04.

**Fig. 4.**
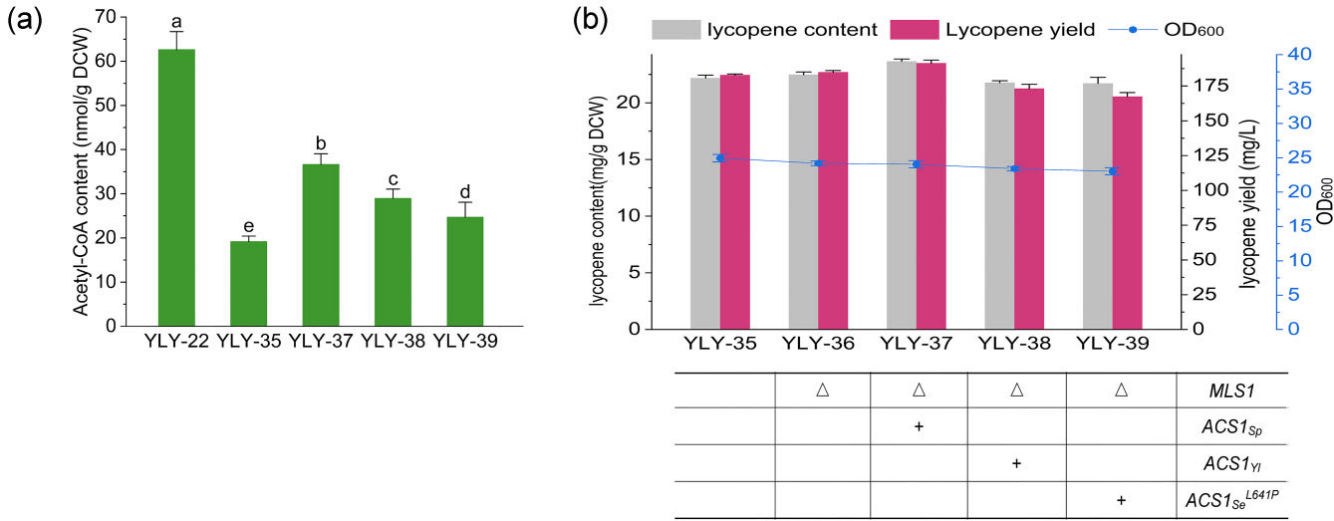
(a) Acetyl-CoA content, (b) the cell growth, lycopene content, and yield of recombinant strains following enhancement of the MVA pathway and acetyl-CoA supply. Note: Different lowercase letters indicated significant differences among different groups: *p *< 0.05. Data are the means ± standard deviations of triplicate experiments.

After increasing the flux through the MVA pathway, we introduced the heterologous acetyl-CoA synthetase genes *ACS1_Sp_, ACS1_Yl_*, and *ACS1_Se_^L641P^* in YLY-35 to investigate the influence on lycopene synthesis, three strains YLY-37 to YLY-39 were thus generated, respectively (Fig. 4). The lycopene content and yield of strain YLY-37 were increased to 23.0 mg/g (DCW) and 185.9 mg/L, with 10.6% and 8.6% higher than those of the reference strain YLY-35, respectively (Fig. 4b). In comparison, the lycopene production of strains YLY-38 and YLY-39 decreased slightly. This confirmed the superior suitability of *ACS1_Sp_* for exogenous acetyl-CoA overproduction in *S. cerevisiae*. It should be noted that the content of acetyl-CoA in YLY-37, YLY-38 and YLY-39 were still higher than that of YLY-35 with 91.0%, 51.0%, and 28.7% increment, respectively, while these values were all significantly decreased compared to YLY-22 strain with only modification of acetyl-CoA synthesis module (Fig. 4a). This implies that modifying the MVA pathway before overexpressing *ACS1* genes is more effective for directing acetyl-CoA into the lycopene pathway and enhancing lycopene production. Nonetheless, due to the amount of acetyl-CoA in strain YLY-37 still higher than YLY-35, the module of lycopene synthesis, namely the downstream pathway of MVA pathway, might become the bottleneck that limiting acetyl-CoA conversion.

### Modulating Heterologous Crt Expression to Increase Lycopene Synthesis

**Fig. 5.**
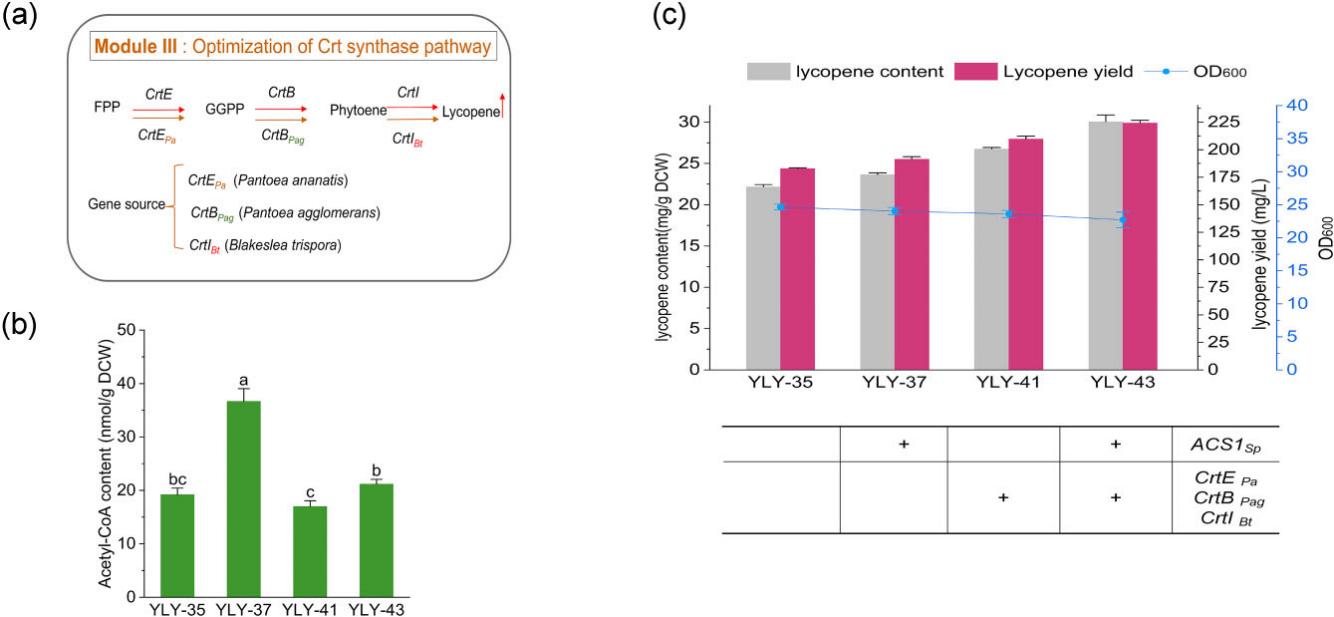
Overexpressing Crt pathway genes to promote lycopene accumulation. (a) Overview of genetic modifications of Module III, (b) the acetyl-CoA content in recombinant strains, and (c) the cell growth, lycopene content, and yield in recombinant strains. Note: Different lowercase letters indicated significant differences among different groups: *p *< 0.05. Data are the means ± standard deviations of triplicate experiments.

To overcome the limitations of the lycopene synthesis pathway, we introduced additional lycopene synthesis pathway by organizing three exogenous enzymes with higher catalytic activity according to the results of previous work (Shi et al., 2019). A combination of *Crt* genes from different species: *CrtE_Pa_* (*Pantoea ananatis*), *CrtB_Pag_* (*Pantoea agglomerans*), and *CrtI_Bt_* (*Blakeslea trispora*) were inserted in strains YLY-35 and YLY-37 to generate the strain YLY-41 and YLY-43, respectively (Fig. 5a). As expected, acetyl-CoA levels continued to decrease in YLY-41 and YLY-43. Particularly, the content in YLY-43 was decreased by 74.4% compared to YLY-37 (Fig. 5b). As a result, the lycopene content and yield in YLY-43 increased to 30.1 mg/g DCW and 224.3 mg/L with an increase of 30.8% and 20.6%, respectively, over YLY-37, which were also 12.6% and 7.3% higher than those of YLY-41 (Fig. 5c). This confirmed the advantage of strengthening lycopene pathway flux for efficient conversion of acetyl-CoA and promoting lycopene synthesis.

### Utilizing Acetate-Repressible Promoters to Down-Regulate *ERG9* Expression

**Fig. 6.**
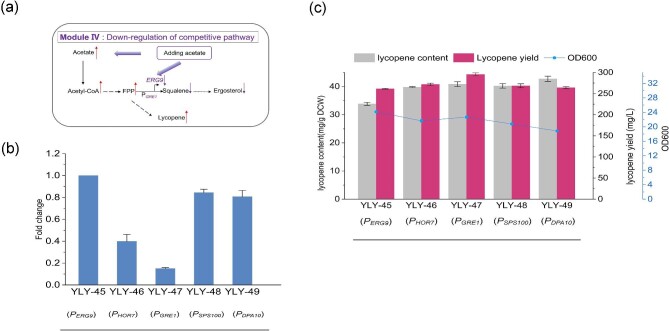
Mining acetate-repressible promoters to reduce *ERG9* expression. (a) Overview of genetic modifications of Module Ⅳ, (b) the changes of relative expression of *ERG9* gene in *ERG9* promoter replacement strains, and (c) the cell growth, lycopene content, and yield in recombinant strains following the addition of 10 g/L acetate. Data are the means ± standard deviations of triplicate experiments.

It is well-established that achieving a rational balance in the competition for precursors FPP between native components (such as ergosterol) and carotenoid synthesis can lead to a noteworthy enhancement in terpenoid products. In this regard, down-regulation of *ERG9* gene which encodes squalene synthase (the first committed step after farnesyl diphosphate in ergosterol biosynthesis), using the repressible promoter is a smart method. Our previous work showed that the addition of 10 g/L acetate can promote lycopene production due to the improvement of intracellular acetyl-CoA concentration (Li et al., 2023). After the addition of 10 g/L acetate, the lycopene content in YLY-43 reached 32.30 mg/g DCW, which was 7.45% higher than that in the control group (without acetate). Due to exogenous acetate inducing toxicity and inhibiting cell growth, it is essential to improve cell tolerance to acetate stress. We simultaneously overexpressed *SET5* and *HST4* in strain YLY-43 to improve cell growth in response to acetate stress and constructed strain YLY-45. Under the condition of adding 10 g/L acetate, the lycopene content of YLY-45 was increased to 33.9 mg/g DCW, which was 4.9% higher than the control YLY-43. Here, aiming to exploit acetate in balancing the metabolic flux of the competition module and lycopene synthesis modules, we tried to mine the acetate-repressible promoter to down-regulate *ERG9* expression levels (Fig. 6a).

According to our transcriptome data under 10 g/L acetate stress (Li et al., 2023), we then mined acetate repressible promoters (Supplementary Table S4), and identified four potential gene promoters (*HOR7, GRE1, SPS100*, and *DPA10*) (Fig. S4). Therefore, we cloned the four genes promoter and replaced the native *ERG9* promoters in strain YLY-45, respectively, and four recombinant strains were generated, including YLY-46 (*HOR7*), YLY-47 (*GRE1*), YLY-48 (*SPS100*), and YLY-49 (*DPA10*) (Supplementary Fig. S5). Subsequently, *ERG9* expression, cell growth, and lycopene synthesis were examined for the reference strain YLY-45 and the four engineered strains. The findings revealed significant suppression of *ERG9* expression in the four engineered strains, with levels reduced to 0.40-fold, 0.15-fold, 0.84-fold, and 0.81-fold compared with the reference strain, respectively (Fig. 6b). Among them, the *GRE1* promoter exhibited the best down-regulation effect (0.15-fold). As expected, the lycopene content of all recombinant strains was increased and the highest increment was observed in strain YLY-47 (*GRE1*) (Fig. 6c). Due to minimal inhibition of cell growth, the lycopene content and yield of strain YLY-47 increased to 40.9 mg/g DCW and 295.5 mg/L, respectively, representing a 20.7% and 13.0% increase over the reference strain YLY-45.

### Engineering NADPH Generation for Enhanced Lycopene Production

**Fig. 7.**
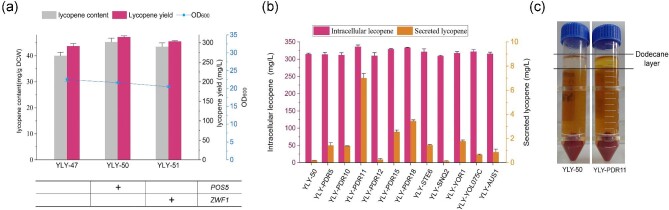
(a) Impact of NADPH synthesis gene overexpression on cell growth, lycopene content, and yield in recombinant strains with the addition of 10 g/L acetate. (b) and (c) Lycopene efflux, intracellular and extracellular lycopene yield of ABC transporter overexpression strains. Data are the means ± standard deviations of triplicate experiments.

NADPH provides the necessary reducing power for lycopene synthesis, as many enzymes in the MVA pathway, such as *tHMG1* and *ERG19*, utilize NADPH as a cofactor (Miziorko, 2011; Ma et al., 2016). However, overproduction of lycopene can deplete the cellular NADPH pool, compromising intracellular redox balance and pathway efficiency. Overexpression of glucose 6-phosphate dehydrogenase (*ZWF1*) and NADH kinase (*POS5*) has been demonstrated as an effective strategy to increase NADPH levels in *S. cerevisiae* (Zhao et al., 2015). We constructed the recombinant strains YLY-50 and YLY-51 by overexpressing *POS5* and *ZWF1* in YLY-47, respectively (Fig. 7a). The growth of the recombinant strains was not affected, while the lycopene content of strains YLY-50 and YLY-51 increased to 45.3 mg/g DCW and 43.5 mg/g DCW, respectively, under the condition of adding 10 g/L acetate, which was 10.8% and 6.3% higher than those of strain YLY-47. The final lycopene yield of the strain YLY-50 reached 314.4 mg/L. Our results demonstrated that overexpression of *POS5* was more effective than overexpression of *ZWF1* for improving lycopene synthesis in *S. cerevisiae*.

### Overexpressing ABC Transporters to Promote Lycopene Efflux

The significant accumulation of carotenoids in microbial cells can induce cytotoxicity, leading to decreased cell growth and reduced production of the target product (Vickers et al., 2014). Promoting the excretion of these substances by engineering endogenous transport system is an effective approach to address this issue (Dunlop et al., 2011)*. Saccharomyces cerevisiae* contains a robust transport system with 11 ABC transporters encoded by *PDR5, PDR10, PDR11, PDR12, PDR15, PDR18, STE6, SNQ2, YOR1, YOL075C*, and *AUS1* (Snider et al., 2013). These ABC transporters provide potential shuttles for efflux carotenoid (Bu et al., 2020). In this study, we upregulated these transporters expression by replacing the original promoters of ABC transporters with inducible *GAL* promoter in YLY-50, respectively, and generated 11 recombinant strains (Supplementary Fig. S6). These strains showed different degrees of lycopene efflux effect. Among them, strain YLY-PDR11 showed the best performance (Fig. 7b). The lycopene efflux level was 12.7 times higher than that of the control strain, reaching 7.3 mg/L, which resulted in the color of the upper n-dodecane organic phase became pale yellow, obviously different from YLY-50 (Fig. 7c). The product efflux also promoted the accumulation of intracellular products, and the highest lycopene content reached 47.9 mg/g DCW (Fig. 7b). Consequently, the intracellular lycopene yield in YLY-PDR11 increased to 336.1 mg/L, 6.9% higher than that of the control strain YLY-50. These results indicated that the Pdr11p is the most potent transporter for efflux lycopene in *S. cerevisiae* cell factory. Future studies could focus on precisely regulating the expression of Pdr11p or specifically engineering the efflux protein to improve the binding capacity and transport efficiency with substrate, further promoting the efflux of lycopene.

## Discussion

The efficient synthesis of carotenoid in recombinant yeast is affected by many factors, primarily including the supply of precursors and cofactors, the catalytic activity of enzymes, and the proximity between enzymes and substrates (Ma et al., 2022; Zhou et al., 2023). The common objective of these strategies is to maximize the carbon source flux toward the carotenoid synthetic pathway, while minimizing flux toward unwanted by-products (Li et al., 2020). However, due to the long synthetic pathway of carotenoids, which involves a multi-step enzymatic reaction, several rate-limiting steps may be formed. Therefore, a multi-modular engineering strategy for carotenoid overproduction is optimal. To date, multi-module metabolic engineering has been successfully applied to the synthesis of a variety of natural products in yeast, such as salidroside, tropane alkaloids, rubusoside, and 7-dehydrocholesterol (Srinivasan & Smolke, 2020; Liu et al., 2021; Xiu et al., 2022; Xu et al., 2022). In this study, a multi-module strategy was applied for lycopene overproduction in *S. cerevisiae*, including sequentially improvement of the precursor acetyl-CoA supply and the metabolic flux of MVA module, and introduction of additional exogenous Crt module. This strategy greatly enhanced the synthesis of lycopene with 3.1-folds increment of product content, and also confirmed the efficiency of multi-module engineering for increasing carotenoid biosynthesis.

Noticeably, our results show that in addition to the selection and number of modules, the modification order of the modules is also a key factor affecting the efficiency of product synthesis. A more significant improvement in lycopene production was achieved by first enhancing the MVA pathway flux before increasing the acetyl-CoA supply. This might be attributed to the fact that modification of one module can easily lead to accumulation of inter-metabolites (sometimes they are toxicity to cells) due to inefficient conversion of these precursors by the downstream module (George et al., 2018), which would restrain cell growth and product synthesis. Therefore, only when the metabolic flux of each module is matched with each other, the maximum lycopene synthesis can be achieved with minimum metabolic burden.

Rational balance of the competition for precursors such as FPP between ergosterol pathway and carotenoid synthesis is essential to achieve high yield of carotenoid in *S. cerevisiae* (Asadollahi et al., 2008). In this study, we developed acetate (the precursor of acetyl-CoA) as a functional signal to balance competing modules and target product modules by using the acetate-repressible promoter *GRE1* to dynamically down-regulate *ERG9* expression levels. The strategy effectively redirected the metabolic flux toward the lycopene pathway, resulting in a 42.3% enhancement in lycopene production with the addition of acetate. The similar growth pattern of the engineered strain compared with the reference strain indicated that the modulation of *ERG9* expression controlled by the *GRE1* promoter does not interfere with cell growth. To further increase carotenoid production, we enhanced the synthesis of cofactors NADPH by overexpressing *POS5*, another limitation factor of carotenoid production, which resulted in 10.8% increment of lycopene content.

Excessive accumulation of intracellular metabolites can lead to cytotoxicity and induce physiological stress (Dunlop et al., 2011). Efflux these toxic products can alleviate the cytotoxicity and further increase the yield of target products. Here, we applied efflux engineering to secret more lycopene by overexpressing 11 endogenous ABC transporter, and found PDR11 is the most efficient transporter for lycopene due to resulted in a 12.7-fold increase of lycopene secretion, which also achieved a 5.8% and 6.9% increase of intracellular lycopene content and yield, respectively. Our study demonstrated that overexpressing the PDR11 transporter increases both extracellular secretion and intracellular accumulation of lycopene, though the efflux quantity remains low with a significant portion retained within the cells. The similar results were reported by Doshi et al., (2013) that overexpressing different ABC transporters in *E. coli* could release small amounts of lycopene. This is likely attributed to their limited substrate affinity and inefficient efflux activity of the transporters (Doshi et al., 2013). To improve efflux efficiency, besides further modification of transport proteins through protein engineering to enhance their affinity and transportation efficiency for lycopene, it's vital to supply additional ATP to strains overexpressing ABC transporters, as ABC transporters are ATP-dependent and function via ATP hydrolysis (Krasowska et al., 2010), such as regulating the expression of energy-producing related genes combined with optimizing fermentation condition. The related work is underway in our lab.

## Conclusion

In summary, we have systematically modified *S. cerevisiae* to improve lycopene production by combined multi-modular metabolic engineering with efflux engineering. To adapt to the heterologous lycopene biosynthetic pathway in yeast, we sequentially modified four modules of lycopene synthesis, including the central carbon pathway for supplying more acetyl-CoA and NADPH, MVA pathway and competitive pathway for diverting more flux to carotenoids synthesis pathway, and introducing an extra lycopene synthesis pathway. We demonstrated the importance of module modification sequence on increasing the synthesis of natural products. Additionally, we found *GRE1* promoter is able to dynamically down-regulate *ERG9* expression in the condition of adding acetate, and redirect the metabolic flux toward the lycopene pathway, thereby enhancing its accumulation. Finally, the efflux engineering strategy was used to reduce the product load of the cells and further increase the production of lycopene. This study provides a new way to promote carotenoid production, which can also be applied in the overproduction of other heterogeneous FPP-derived hydrophobic compounds with similar synthesis and storage pattern.

## Supplementary Material

kuae015_Supplemental_File

## Data Availability

All data generated or analyzed during this study are included in this manuscript and its additional file.
