# Supplementary material for: Multi-modular metabolic engineering and efflux engineering for enhanced lycopene production in recombinant Saccharomyces cerevisiae
Source: J Ind Microbiol Biotechnol. 2024 Apr 15;51:kuae015. doi: 10.1093/jimb/kuae015 (PMC11074996; doi:10.1093/jimb/kuae015)
Supplement: kuae015_Supplemental_File [file kuae015_supplemental_file.docx]

**Title:** Multi-modular metabolic engineering and efflux engineering for enhanced lycopene production in recombinant *Saccharomyces cerevisiae*

**Authors:** Guangxi Huang^1,2^, Jiarong Li^1,2^, Jingyuan Lin^1,2^, Changqing Duan^1,2^, Guoliang Yan^1,2,3 *^

**Affiliated Address:**

1. Centre for Viticulture and Enology, College of Food Science and Nutritional Engineering, China Agricultural University, Beijing 100083, China
2. Key Laboratory of Viticulture and Enology, Ministry of Agriculture and Rural Affairs, Beijing 100083, China
3. Beijing Key Laboratory of Functional Food from Plant Resources, College of Food Science and Nutritional Engineering, China Agricultural University, Beijing 100083, China

**File type:** Supplementary information

***Corresponding author:** Guoliang Yan

Tel: +86-10-62737039; Fax: +86-10-62738658; E-mail: [glyan@cau.edu.cn](mailto:glyan@cau.edu.cn)

17 East Tsinghua Rd, College of Food Science and Nutritional Engineering, China Agricultural University, Beijing, 100083, China

**Supplementary tables:**

**Table S1** Plasmids used in this study

| Plasmid | Description | Source |
| --- | --- | --- |
| pUMRI-21 | No Homologus Arm（HA）;  loxp-KanMX-URA3-pbr322ori-loxp，  TADH1-MCS1-PGAL10-PGAL1-MCS2-TCYC1 | (Lv et al., 2016) (KM216411) |
| pLY-*CrtI*-*CrtB* -*GAL1-10-7* | pUMRI-21, GAL1-10-7 HA,  TADH1-CrtI-PGAL10-PGAL1-CrtB-TCYC1 | This study |
| pLY-*CrtE*-*GAL80* | pUMRI-21, *GAL80* HA, T*ADH1*-*CrtE*-P*GAL10*-P*GAL1*-*MCS2*-T*CYC1* | This study |
| pLY-*YPL062W* | pUMRI-2, *YPL062W* HA | This study |
| pLY-*ACS2*-*YPL062W* | pUMRI-21, *YPL062W* HA, T*ADH1*-*ACS2*-P*GAL10*-P*GAL1*-*MCS2*-T*CYC1* | This study |
| pLY*-MLS1* | pUMRI-21, *MLS1* HA | This study |
| pLY-*ACS1SP-MLS1* | pUMRI-21, *MLS1* HA, T*ADH1*-*MCS1*-P*GAL10*-P*GAL1*-*ASC1SP*-T*CYC1* | This study |
| pLY- *ACS1YL -MLS1* | pUMRI-21, *MLS1* HA, T*ADH1*-*MCS1*-P*GAL10*-P*GAL1*- *ACS1YL*-T*CYC1* | This study |
| pLY-*ACS1SE L641P -MLS1* | pUMRI-21, *MLS1* HA, T*ADH1*-*MCS1*-P*GAL10*-P*GAL1*- *ACS1SE L641P* -T*CYC1* | This study |
| pLY*-CIT2* | pUMRI-21, *CIT2* HA | This study |
| pLY-*ERG10 -CIT2* | pUMRI-21, *CIT2* HA, T*ADH1*-*ERG10*-P*GAL10*-P*GAL1*-*MCS2*-T*CYC1* | This study |
| pLY-*ERG12 -CIT2* | pUMRI-21, *CIT2* HA, T*ADH1*-*ERG12*-P*GAL10*-P*GAL1*-*MCS2*-T*CYC1* | This study |
| pLY-*ERG13 -CIT2* | pUMRI-21, *CIT2* HA, T*ADH1*-*ERG13*-P*GAL10*-P*GAL1*-*MCS2*-T*CYC1* | This study |
| pLY-*ERG20 -CIT2* | pUMRI-21, *CIT2* HA, T*ADH1*-*ERG20*-P*GAL10*-P*GAL1*-*MCS2*-T*CYC1* | This study |
| pLY-*tHMG1 -CIT2* | pUMRI-21, *CIT2* HA, T*ADH1*-*tHMG1*-P*GAL10*-P*GAL1*-*MCS2*-T*CYC1* | This study |
| pLY*-ROX1* | pUMRI-21, *ROX1* HA | This study |
| pLY-*ERG12 -ROX1* | pUMRI-21, *ROX1* HA, T*ADH1*-*ERG12*-P*GAL10*-P*GAL1*-*MCS2*-T*CYC1* | This study |
| pLY-*ERG12-ERG20-* ROX1 | pUMRI-21, *ROX1* HA, T*ADH1*-*ERG12*-P*GAL10*-P*GAL1*-*ERG20*-T*CYC1* | This study |
| pLY*-DOS2* | pUMRI-21, *DOS2* HA, | This study |
| pLY-*tHMG1-DOS2* | pUMRI-21, *DOS2* HA, T*ADH1*-*tHMG1*-P*GAL10*-P*GAL1*-*MCS2*-T*CYC1* | This study |
| pLY-*CrtI-HO* | pUMRI-21, *HO* HA, T*ADH1*-*CrtI*-P*GAL10*-P*GAL1*-*MCS2*-T*CYC1* | This study |
| pLY-*CrtB-CrtE-PAH1* | pUMRI-21, *PAH1* HA, T*ADH1*-*CrtB*-P*GAL10*-P*GAL1*-*CrtE*-T*CYC1* | This study |
| pLY-*HOR7* | The promoter (*HOR7*), *ERG9* R, and *ERG9* F were ligated together by overlap method, then infused with PuvII/BssHII digested pUMRI-21 plasmid which contains the loxp-kanMX-URA3-pbr322ori-loxp region | This study |
| pLY-*GRE1* | The promoter (*GRE1*), *ERG9* R, and *ERG9* F were ligated together by overlap method, then infused with PuvII/BssHII digested pUMRI-21 plasmid which contains the loxp-kanMX-URA3-pbr323ori-loxp region | This study |
| pLY-*SPS100* | The promoter (*SPS100*), *ERG9* R, and *ERG9* F were ligated together by overlap method, then infused with PuvII/BssHII digested pUMRI-21 plasmid which contains the loxp-kanMX-URA3-pbr324ori-loxp region | This study |
| pLY-*DPA10* | The promoter (*DPA10*), *ERG9* R, and *ERG9* F were ligated together by overlap method, then infused with PuvII/BssHII digested pUMRI-21 plasmid which contains the loxp-kanMX-URA3-pbr325ori-loxp region | This study |
| pBX-*PDR5* | The promoter P_GAL1_（*PDR5*）, *PDR5* R, and *PDR5* F were ligated together by overlap method, then infused with PuvII/BssHII digested pUMRI-21 plasmid which contains the loxp-kanMX-URA3-pbr325ori-loxp region | This study |
| pBX-*PDR10* | The promoter P*GAL1*（*PDR10*）, *PDR10* R, and *PDR10* F were ligated together by overlap method, then infused with PuvII/BssHII digested pUMRI-21 plasmid which contains the loxp-kanMX-URA3-pbr325ori-loxp region | This study |
| pBX-*PDR11* | The promoter P*GAL1*（*PDR11*）, *PDR11*R, and *PDR11* F were ligated together by overlap method, then infused with PuvII/BssHII digested pUMRI-21 plasmid which contains the loxp-kanMX-URA3-pbr325ori-loxp region | This study |
| pBX-*PDR12* | The promoter P*GAL1*（*PDR12*）, *PDR12* R, and *PDR12* F were ligated together by overlap method, then infused with PuvII/BssHII digested pUMRI-21 plasmid which contains the loxp-kanMX-URA3-pbr325ori-loxp region | This study |
| pBX-*PDR15* | The promoter P*GAL1*（*PDR15*）, *PDR15* R, and *PDR15* F were ligated together by overlap method, then infused with PuvII/BssHII digested pUMRI-21 plasmid which contains the loxp-kanMX-URA3-pbr325ori-loxp region | This study |
| pBX-*PDR18* | The promoter P*GAL1*（*PDR18*）, *PDR18* R, and *PDR18* F were ligated together by overlap method, then infused with PuvII/BssHII digested pUMRI-21 plasmid which contains the loxp-kanMX-URA3-pbr325ori-loxp region | This study |
| pBX-*STE6* | The promoter P*GAL1*（*STE6*）, *STE6* R, and *STE6* F were ligated together by overlap method, then infused with PuvII/BssHII digested pUMRI-21 plasmid which contains the loxp-kanMX-URA3-pbr325ori-loxp region | This study |
| pBX-*SNQ2* | The promoter P*GAL1*（*SNQ2*）, *SNQ2* R, and *SNQ2* F were ligated together by overlap method, then infused with PuvII/BssHII digested pUMRI-21 plasmid which contains the loxp-kanMX-URA3-pbr325ori-loxp region | This study |
| pBX-*YOR1* | The promoter P*GAL1*（*YOR1*）, *YOR1* R, and *YOR1* F were ligated together by overlap method, then infused with PuvII/BssHII digested pUMRI-21 plasmid which contains the loxp-kanMX-URA3-pbr325ori-loxp region | This study |
| pBX-*YOL075C* | The promoter P*GAL1*（*YOL075C*）, *YOL075C* R, and *YOL075C* F were ligated together by overlap method, then infused with PuvII/BssHII digested pUMRI-21 plasmid which contains the loxp-kanMX-URA3-pbr325ori-loxp region | This study |
| pBX-*AUS1* | The promoter P*GAL1*（*AUS1*）, *AUS1* R, and *AUS1* F were ligated together by overlap method, then infused with PuvII/BssHII digested pUMRI-21 plasmid which contains the loxp-kanMX-URA3-pbr325ori-loxp region | This study |

All plasmids share the same structure of *loxp-kanMX-URA3-pbr322ori-loxp*.

**Table S2** Primers used in this study

| **Primers** | Sequences (5’-3’) | Note |
| --- | --- | --- |
| **For amplification of structural genes** | | |
| ERG10 F | GTAAGAATTTTTGAAAATTCATGTCTCAGAACGTTTACATTGTAT | ERG10 |
| ERG10 R | GCCGCCCTTTAGTGAGGGTTTCATATCTTTTCAATGACAATAGAGGAAG |  |
| ERG12F | GTAAGAATTTTTGAAAATTCATGTCATTACCGTTCTTAACTTCTG | ERG12 |
| ERG12 R | GCCGCCCTTTAGTGAGGGTTTTATGAAGTCCATGGTAAATTCGTG |  |
| ERG13 F | GTAAGAATTTTTGAAAATTCATGAAACTCTCAACTAAACTTTGTTG | ERG13 |
| ERG13 R | GCCGCCCTTTAGTGAGGGTTTTATTTTTTAACATCGTAAGATCTTC TAAATTTGT |  |
| ERG20 F | GTAAGAATTTTTGAAAATTCATGGCTTCAGAAAAAGAAATTAGGA | ERG20 |
| ERG20 R | GCCGCCCTTTAGTGAGGGTTCTATTTGCTTCTCTTGTAAACTTTG |  |
| tHMG1 F | GTAAGAATTTTTGAAAATTCGACCAATTGGTGAAAACTGAAGT | tHMG1 |
| tHMG1 R | GCCGCCCTTTAGTGAGGGTTTTAGGATTTAATGCAGGTGACG |  |
| CrtE F | CGTCAAGGAGAAAAAACCCCATGGCTTATACCGCAATGG | CrtE |
| CrtE R | CCTATAGTGAGTCGTATTACTTAGTTTTGCCTGAAAGCGATG |  |
| CrtI F | GTAAGAATTTTTGAAAATTCATGTCCGACCAGAAGAAGC | CrtI |
| CrtI R | ATAGTGAGTCGTATTACCTATTTGCTTCTCTTGTAAACT |  |
| CrtB F | AATTCAACCCTCACTAAAGGATGTCCCAACCACCATTG | CrtB |
| CrtB R | TGTAATCCATCGATACTAGTTCAAACTGGTCTTTGCCAAAG |  |
|  | **For amplification of homologous arms** |  |
| YPL062W FF | ATATTCAATTCGAAGTGTTCAGTCGGCCATATAGGCCTTCATTCGAATATAAGGCCGC | YPL062W  homologous arm |
| YPL062W FR | AACAAAAGCTGGAGCTGGCCTTATGTCGAAAAACAGGCCA |  |
| YPL062W RF | GGCGTAATAGCGAAGAGGCCCATTGATCTCCTCTTGGGAAC |  |
| YPL062W RR | GCGGCCTTATATTCGAATGAAGGCCTATATGGCCGACTGAACACTTCGAATTGAATAT |  |
| CIT2 FF | CGGTGCTTCCATTGAAAGGGGCCTATATGGCCCATGCTCTCAAAAGGGCC | CIT2 homologous arm |
| CIT2 FR | AACAAAAGCTGGAGCTGGTGAATGTCGGCGATCGTAC |  |
| CIT2 RF | GGCGTAATAGCGAAGAGGCCGGGATACTCTAAACTCAGGAAGAG |  |
| CIT2 RR | GGCCCTTTTGAGAGCATGGGCCATATAGGCCCCTTTCAATGGAAGCACCG |  |
| DOS2 FF | GATGCACAGGAGGAAGACAGGCCATATAGGCCACGGAGTCACTAGTGTCATT | MLS1  homologous arm |
| DOS2 FR | AACAAAAGCTGGAGCTGGTATCCGTCATCTTTCTCGATTACTA |  |
| DOS2 RF | GGCGTAATAGCGAAGAGGCCTGTTCTATTTTACAGGGCGACA |  |
| DOS2 RR | AATGACACTAGTGACTCCGTGGCCTATATGGCCTGTCTTCCTCCTGTGCATC |  |
| ROX1 FF | AACTCAAGCAACACTGAGGTGGCCTATATGGCCAAGAAAAGGGCCTATTGTTGC | MLS1  homologous arm |
| ROX1 FR | AACAAAAGCTGGAGCTGGGGGTCTTGGAATCTTAGGTGT |  |
| ROX1 RF | GGCGTAATAGCGAAGAGGCCTGGTCTCCAGATCTTTAAGTGGAC |  |
| ROX1 RR | GCAACAATAGGCCCTTTTCTTGGCCATATAGGCCACCTCAGTGTTGCTTGAGT |  |
| MLS1 FF | AAGAACAAGTGGAAAGACTGTCTAGGCCTATATGGCCTACACTGGCTACCGATTTAAC | MLS1  homologous arm |
| MLS1 FR | AACAAAAGCTGGAGCTGGTTTGCAGTTTCAGGCAGGAA |  |
| MLS1 RF | GGCGTAATAGCGAAGAGGCCAGCCAATGAAAAGGCCAT |  |
| MLS1 RR | AGTTAAATCGGTAGCCAGTGTAGGCCATATAGGCCTAGACAGTCTTTCCACTTGTTCTT |  |
|  | **For promoter replacement plasmids construction** |  |
| ERG9 FF | CACGACTACGACGTGTACTGGGATCCGGTCTGCAGGGGAGAACTTA | ERG9 F |
| ERG9 FR | GTTATATTAAGGGTTGTCGAGCGCGCAAAACCGATAACGCCTTCC |  |
| ERG9 RR | TAAGTTCTCCCCTGCAGACCGGATCCCAGTACACGTCGTAGTCGTG | ERG9 R（HOR7） |
| ERG9 RF （HOR7） | TATCTCAAGTTGTTGTTTCCGCCACACAATGGGAAAGCTATTACAA |  |
| HOR7 F | TAGGCCTCTTCGCTATTACGCCAGCTCGGATATACTCGCTCGC | HOR7 promoter |
| HOR7 R | TTGTAATAGCTTTCCCATTGTGTGGCGGAAACAACAACTTGAGATA |  |
| ERG9 RF （GRE1） | GCTGATAAGTTGCACGGCCACACAATGGGAAAGCTATTACAA | ERG9 R（GRE1） |
| GRE1 F | TAGGCCTCTTCGCTATTACGCCAGTCTTGATGAAGTGGGACGC | GRE1 promoter |
| GRE1 R | TTGTAATAGCTTTCCCATTGTGTGGCCGTGCAACTTATCAGC |  |
| ERG9 （SPS100）RF | TTCTTGCAACTTTAACAGCTTCTGCACACAATGGGAAAGCTATTACAA | ERG9 R（SPS100） |
| SPS100 F | TAGGCCTCTTCGCTATTACGCCAGATCCCCAAAAATGATAGCATTTG | SPS100  promoter |
| SPS100 R | TTGTAATAGCTTTCCCATTGTGTGCAGAAGCTGTTAAAGTTGCAAGAA |  |
| ERG9 （DPA10）RF | GCAAACAACTGCCAAGTGCACACAATGGGAAAGCTATTACAA | ERG9 R（DPA10） |
| DPA10 F | TAGGCCTCTTCGCTATTACGCCAGGGAACACTGTCGCAATG | DPA10 promoter |
| DPA10 R | TTGTAATAGCTTTCCCATTGTGTGCACTTGGCAGTTGTTTGC |  |
| POS5 F | CGTCAAGGAGAAAAAACCCCATGTTTGTCAGGGTTAAATTGAATAAAC | POS5 |
| POS5 R | CCTATAGTGAGTCGTATTACTTAATCATTATCAGTCTGTCTCTTGGT |  |
| ZWF1 F | CGTCAAGGAGAAAAAACCCCATGGATGGTCCCAATTTTGC | ZWF1 |
| ZWF1 R | CCTATAGTGAGTCGTATTACTCATACAAGTTTATCAACCCAAGAGAC |  |
| GAL1 F* | CCTCTTCGCTATTACGCCAGTGAAGTACGGATTAGAAGCCG |  |
| GAL1 R （PDR5） | TTATTGTTAAGCTTGGCCTCGGGCATCTCCTTG ACGTTAAAGTATAGAGG | P*GAL1*（*PDR5*）promoter |
| GAL1 R（PDR10） | TTATTGTTAAGCTTGGCCTCGGGCATCTCCTTG ACGTTAAAGTATAGAGG | P*GAL1*（*PDR10*）promoter |
| GAL1 R （PDR11） | ATATTTGGAAAGAGACATCCGCCTCCTTGACGTTAAAGTATAGAGG | P*GAL1*（*PDR11*）promoter |
| GAL1 R （PDR12） | TGTTCGTCAGTCGAAGACATCTCCTTGACGTTAAAGTATAGAGG | P*GAL1*（*PDR12*）promoter |
| GAL1 R （PDR15） | TCTACGTCTCTGATATCTGATGACATTTTTCTCCTTGACGTTAAAGTATAGAG | P*GAL1*（*PDR15*）promoter |
| GAL1 R （PDR18） | CTTCTACTGAAACGCATTCCATTCTCCTTGACGTTAAAGTATAGAGG | P*GAL1*（*PDR18*）promoter |
| GAL1 R （SNQ2） | CGTGCTTTTGATATTGCTCATTTTTCTCCTTGACGTTAAAGTATAGAG | P*GAL1*（*SNQ2*）promoter |
| GAL1 R （STE6） | TGTTTTGTAGTCTTAAAACTTAAAAAGTTCATTCTCCTTGACGTTAAAGTATAGAGG | P*GAL1*（*STE6*）promoter |
| GAL1 R （YOR1） | TCCCCCACGGTAATCGTCATCTCCTTGACGT TAA  AGTATAGAGG | P*GAL1*（*YOR1*）promoter |
| GAL1 R （YOL075C） | CCATTCTCCTGCTGTGACATATCTCCTTGACGTTAAAGTATAGAGG | P*GAL1*（*YOL075C*）promoter |
| GAL1 R（AUS1） | GAGTGAAGTACTTTGAAATTGACATCTCCTTGACGTTAAAGTATAGAGG | P*GAL1*（*AUS1*）promoter |
| PDR5 FF | GGTAGACCAGGCTCTGGCTGTACTGGATCCTTGGCGCAGTCCCTTACATAGT | PDR5 F |
| PDR5 FR | GTTATATTAAGGGTTGTCGAGCGCGCAGAGT TCCGCGGAGACATTTC |  |
| PDR5 RF | CCTCTATACTTTAACGTCAAGGAGATGCCCGGGCCAAGCTTAACAATAA | PDR5 R |
| PDR5 RR | ACTATGTAAGGGACTGCGCCAAGGATCCAGTACAGCCAGAGCCTGGTCTACC |  |
| PDR10 FF | GAGTTGGAGGTGCATATCCCGACGAATTCCGCC TGAGTTACTCTCCTCTGG | PDR10 F |
| PDR10 FR | GTTATATTAAGGGTTGTCGAGCGCGCCGGGCGGC TGTAACAATGAC |  |
| PDR10 RF | CCTCTATACTTTAACGTCAAGGAGAATGTTGCAA GCGCCCTCAAG | PDR10 R |
| PDR10 RR | CCAGAGGAGAGTAACTCAGGCGGAATTCGTCGG GATATGCACCTCCAACTC |  |
| PDR11 FF | GGTGAAAGGAAACGTATCTCCGGATCCTCCACTTTGACGCCCCTTTA | PDR11 F |
| PDR11 FR | ATATTAAGGGTTGTCGAGCGCGCAGAGAGCTTACGATGTTG |  |
| PDR11 RF | CCTCTATACTTTAACGTCAAGGAGGCGGATGTCT CTTTCCAAATAT | PDR11 R |
| PDR11 RR | TAAAGGGGCGTCAAAGTGGAGGATCCGGAGATACGTTTCCTTTCACC |  |
| PDR12 FF | CAAAATTGTACGGGTGTCGTGGATCCAGTGG CCTCTAAACCAAAGAT | PDR12 F |
| PDR12 FR | ATATTAAGGGTTGTCGAGCGCGCATAAGAA CCG TCGAGAAAATGT |  |
| PDR12 RF | CCTCTATACTTTAACGTCAAGGAGATGTCTT CGA CTGACGAACA | PDR12 R |
| PDR12 RR | ATCTTTGGTTTAGAGGCCACTGGATCCACGA CACCCGTACAATTTTG |  |
| PDR15 FF | GCCGATGTGTCATACCAGTCGGATCCTGGCT GTAGAACATGCCTTAC | PDR15 F |
| PDR15 FR | ATATTAAGGGTTGTCGAGCGCGCTACAACA AGACTAGCAAATCTCT |  |
| PDR15 RF | CCTCTATACTTTAACGTCAAGGAGAAAAATGTCA TCAGATATCAGAGACGTAGA | PDR15 R |
| PDR15 RR | GTAAGGCATGTTCTACAGCCAGGATCCGACTGGT ATGACACATCGGC |  |
| PDR18 FF | ACTGCCAATAGAGAATTCTATGCGGATCCGACTCACCT GGTAAAACATCTATG | PDR18 F |
| PDR18 FR | ATATTAAGGGTTGTCGAGCGCGGTGTAAAATACAGAATTCCCGG |  |
| PDR18 RF | CCTCTATACTTTAACGTCAAGGAGAATGGAATGC GTTTCAGTAGAAG | PDR18 R |
| PDR18 RR | CATAGATGTTTTACCAGGTGAGTCGGATCCGCAT  AGAATTCTCTATTGGCAGT |  |
| SNQ2 FF | GAGTGCCCTAGAAGGTGCTAGGATCCGCCAGACT ATGTATGACTCG | SNQ2 F |
| SNQ2 FR | GTTATATTAAGGGTTGTCGAGCGCGCTCTCAGGG AGCTTGAACTCTA |  |
| SNQ2 RF | CTCTATACTTTAACGTCAAGGAGAAAAATGA GCA ATATCAAAAGCACG | SNQ2 R |
| SNQ2 RR | CGAGTCATACATAGTCTGGCGGATCCTAGCA CCTTCTAGGGCACTC |  |
| STE6 FF | ATTTAGTTGCAATATGTGCGCGGATCCCTAT  GACTTCCATAGCTCACC | STE6 F |
| STE6 FR | ATATTAAGGGTTGTCGAGCGCGTGCCCTCTG TGG GAATTGAAC |  |
| STE6 RF | CCTCTATACTTTAACGTCAAGGAGAATGAACTTT  TTAAGTTTTAAGACTACAAAACA | STE6 R |
| STE6 RR | GGTGAGCTATGGAAGTCATAGGGATCCGCG CAC  ATATTGCAACTAAAT |  |
| YOR1 FF | GAACGATACAGCCGAACGATCTCGGATCCGGAA  GAAGGAAGTTTAGTGCCACC | YOR1 F |
| YOR1 FR | ATATTAAGGGTTGTCGAGCGCGGCGGAGAG CTG  TTCTACCTCC |  |
| YOR1 RF | CCTCTATACTTTAACGTCAAGGAGATGACGATTA  CCGTGGGGGA | YOR1 R |
| YOR1 RR | GGTGGCACTAAACTTCCTTCTTCCGGATCCGAGATCGTTCGGCTGTATCGTTC |  |
| YOL075C FF | CTCTTCTGAGCGAACCAAGAAGGATCCACCATTG GAGAATACCGTTTG | YOL075C F |
| YOL075C FR | ATATTAAGGGTTGTCGAGCGCGCTTGTGCCTACTT CTGCATT |  |
| YOL075C RF | CCTCTATACTTTAACGTCAAGGAGATATGTCACAGCAGGAGAATGG | YOL075C R |
| YOL075C RR | CAAACGGTATTCTCCAATGGTGGATCCTTCTTGGTTCGCTCAGAAGAG |  |
| AUS1 FF | AATTCGGCCTGTCTCATGTAGGATCCAAAGTAAG  CCATACGAACGG | AUS1 F |
| AUS1 FR | ATATTAAGGGTTGTCGAGCGCGAGAACTGTTCAGTGCCTTAGA |  |
| AUS1 RF | CCTCTATACTTTAACGTCAAGGAGATGTCAATTTCAAAGTACTTCACTC | AUS1 R |
| AUS1 RR | CCGTTCGTATGGCTTACTTTGGATCCTACATGAGACAGGCCGAATT |  |
|  | **For quantitative RT-PCR** |  |
| qACT1-F | GAAATGCAAACCGCTGCTCA | 145bp |
| qACT 1-R | TACCGGCAGATTCCAAACCC |  |
| qDPA10-F | ACTGCCAAGTGTTATGTCCCA | 112bp |
| qDPA10-R | ACCTCTTCGTTGTCAGTGCAAA |  |
| qGRE1-F | GCACTGGTGGTGGCACTTAT | 132bp |
| qGRE1-R | TCCTGACCCAGACAGATCGC |  |
| qSPS100-F | CGGGTGGACCTGCTGTATCT | 123bp |
| qSPS100-R | GGAACGCCAGTTTGTGACGA |  |
| qHOR7-F | TCAAGTTGTTGTTTCCGCCG | 108bp |
| qHOR7-R | CCAAAGCACCAGCTAGAGCAA |  |
|  | **For genomic PCR confirmation** |  |
| YZ GAL1-10-7 F1 | AACTTCAACAGAGCCTAAAAT | 969 |
| YZ CrtI R1 | TCGGGAAGTCCAAGCT |  |
| YZ CrtB F1 | TGCGGTGTGAAATACCGC | 1107 |
| YZ GAL1-10-7 R1 | AAAGGTTTGCCAGTGCTCC |  |
| YZ CrtE F1 | CGGCTACACTAGAAGGACA | 1054 |
| YZ GAL80 R1 | CGGAAAACTTGCGCCATTTAAA |  |
| YZ HOR7 F | AGGGGCTACAGTCTCGATAA | 1100bp |
| YZ DPA10 F | TATCGAATTCCTTTAGGCCGT | 1073bp |
| YZ GRE1 F | TCCCTCTTTAACCACAGTTCT | 953bp |
| YZ SPS100 F | TCGCCGAAAGAAGCTAAG | 1066bp |
| YZ ERG9 R* | GTAGGAAAAGACCCATGCT |  |
| YZ GAL1 F* | TTCCTGAAACGCAGATGTGC |  |
| YZ PDR5 R | GATGTACATCAGCTTCTGCG | 931 |
| YZ PDR10 R | TCAGCAGCGTAGTACATCCT | 945 |
| YZ PDR11 R | ACCGATCTTGTAGCTTTTGC | 961 |
| YZ PDR12 R | ACCGATCTTGTAGCTTTTGC | 1106 |
| YZ PDR15 R | CCATCCATGGGTTTCAGGAT | 1069 |
| YZ PDR18 R | CCTTTCGCTGCTAATGCTTC | 1032 |
| YZ SNQ2 R | CCAAAATCATTTCACCCGCT | 1021 |
| YZ STE6 R | GGCTGCTTTACTCGTTTCAG | 1064 |
| YZ YOR1 R | CCTCTTCTTCTGTCGCTTCTG | 1034 |
| YZ YOL075C R | GTCTTCTCTTTTCACCACCAG | 1102 |
| YZ AUS1 R | GAGAGCTGTGGCGGAATCTA | 1206 |

**Table S3 Sequence of heterologous genes**

| **Original source of genes** | **Codon-optimized sequence** |
| --- | --- |
| ACS1_Sp_ | ATGGGACATCATGTGACCGGGCTACCCGATAGCATCGACCCTTCCGAAGACGTTTTTCCTCCCCCAAAAAGGGTCCAGGGTGGACATAATGGCTGTCCAAAACCACACATAGGACCCGATTACGAAAGCTATTTGAAGGAGTATAAAAAGACAGTTGGGCCCGACGCGGACAAATGGTGGGCAGAGCAAGCAAATCAGCACCTAGATTGGATCAGGCCCTTTAAGACAGTTAGAGCAGGAGGTTTCGAATATGGGGACGTCCAGTGGTTTGTAGAGGGACAACTAAATGCGGCCTACAATTGTTTGGATAGACATTTCTACGCGCATCCCGACAAGACCGCCATTATCTTTGAAGCCGACGAAAGCGGTGACTCAAGAGAAATTTCCTGGGGAGAGCTTATGAGAGAAACATGCAGAGTGGCGAATGTACTTAAGGAGTGGGGGGTCAAGAAGGGGGACGCCGTGTCAATTTATTTGCCCATGACATGGCAGGCAGCGGCTGCCTTCTTGGCATGCGCGCGTGTGGGGGCAGTCCACTCAGCAGTTTTCGCCGGATTTAGCGCGGAAAGTTTGAGAGACAGGGTGAACGATTGCGAATGCAAGGTTTTGATTACCTCAGATGAAGGCAAACGTGGCGGCAAGACAATCGCAACCAAAGCTATTGTTGACGCGGCTTTAGCGCAATGCCCTATGGTAGAACATGTCTTGGTTCTTAGAAGAACTGGGGGTAAGGTAAATATGAAGGAAGGTAGAGACTCATGGTGGGACGAGGAATGTGCAAAAGTGCCAACATATTGTCCTTGTGAACCAATGAGTGCAGAAGACCCTCTGTTCATTCTTTACACGAGCGGGTCAACTGGAAAACCAAAAGGAGTGGTACACAGTACAGGCGGGTACTTATTGTGTGCATTGATAACAGTTAAGTATGTATTTGATGTTCATCCCGGAGACAAATTTGCATGTATGGCAGATGTAGGGTGGATAACCGGCCACACGTATATCGTATACGGACCGCTAGCGCAAGGAGTAACCACAACAATCTTTGAATCAACACCCGTGTATCCTACCGCCTCAAGGTACTGGGACTTTGTGGATAAATGGAAAGCGACGCACTTGTACACAGCTCCGACCGCGATCAGATTGCTTAGGAGGATGGGGGAAGAGCACGTGAAGCATCATGATCTATCCTCTCTTAGGGTTTTAGGCACAGTCGGAGAACCGATAAATCCTGAGGCGTGGCATTGGTACAATGACTTCGCTGGGAAAAACCAGTGCGCGATAGTAGACACCTACTGGATGACCGAAACCGGATCTCATGCGGTGGCCCCCTATCCGGGTGCCATTCACACGAAACCAGGTTCAGCAACGTTCCCTTGTTTCGGCTTTGACTTAGCCATCATAGACCCACAGACAGGTAAGGAGTTAGAAGGCAACGACGTGGAAGGAGTTCTGGCAGCCCGTGCTCCTTGGCCCAGCCTTGCCAGAACCGTTTTTAGGGACCACAAGAGATACCTAGAAACATATATGAAACCTTATCCCGGATATTTTTTTTTCGGTGATGGTGCAGCACGTGATGCAGACGGATACCTTTGGATTAAAGGGAGGGTTGATGATGTCATTAATGTATCAGGTCACAGATTATCTACTGCGGAAGTCGAATCAGCGTTGATACTACATAAAGGCGTGGCAGAAACTGCTGTCGTAGGTTCACATGATGATATCACGGGACAAGCAGTGTATGCTTTTGTCACGATGAAGCCGGAGTTTGATCTAGAGGCTACAAAAGAAGATGCGCTGGCCAAGGAGTTGGCGATACAAGTTAGAAAAGTAATCGGACCTTTTGCGGCACCCAAGAAGATATATTTAGTAAGTGACCTTCCGAAGACCAGGTCTGGTAAAATTATGAGGAGAATATTAAGAAAGGTAGTCGCAGGAGAAGGTGACCAATTGGGCGACTTGTCCTCCATAGCTGATCCAAGCATTGTCGACGAGATAAAGAGCAAAGTGGCCGGTAAATGA |
| ACS1_Yl_ | ATGTCTGAGGATCATCCAGCAATCCATCCGCCGTCTGAATTCAAGGACAACCACCCACATTTCGGAGGTCCACATTTGGATTGTTTACAAGATTACCACCAACTGCACAAGGAGTCAATAGAAGATCCTAAGGCATTCTGGAAGAAGATGGCAAATGAACTTATCTCATGGAGTACGCCATTCGAAACCGTTCGTTCAGGAGGATTCGAACACGGGGATGTAGCTTGGTTCCCTGAGGGACAGTTAAATGCCAGCTACAACTGCGTCGACCGTCATGCGTTTGCCAACCCCGATAAGCCAGCAATTATTTTTGAAGCAGATGAGCCCGGACAAGGCCGTATAGTAACCTACGGGGAATTACTTAGGCAGGTTAGTCAAGTTGCTGCTACTCTTCGTTCCTTTGGTGTCCAAAAGGGAGATACAGTTGCAGTTTATTTGCCTATGATACCGGAAGCGATTGTAACCCTGTTAGCTATCACGAGAATTGGAGCCGTACACTCTGTAATATTTGCAGGGTTCAGCAGTGGCTCTCTGAGAGACAGAATCAATGATGCTAAAAGTAAGGTGGTTGTGACTACTGACGCTTCAATGCGTGGCGGAAAGACCATCGATACCAAAAAAATTGTGGACGAGGCATTGAGAGACTGTCCGTCAGTAACACACACACTAGTCTTTAGAAGGGCGGGCGTCGAAAATCTAGCCTGGACAGAAGGTAGGGACTTCTGGTGGCATGAAGAGGTTGTGAAGCACAGGCCATATTTGGCACCCGTTCCTGTTGCTAGTGAAGACCCCATCTTCCTGCTATACACATCTGGCAGTACCGGCACACCCAAAGGTCTTGCTCACGCTACGGGGGGGTATTTATTAGGTGCTGCGTTGACAGCTAAGTATGTTTTTGACATACACGGAGACGACAAGTTATTCACAGCGGGGGACGTTGGATGGATCACTGGCCACACGTATGTATTATACGGTCCACTTATGCTAGGTGCAACCACTGTAGTTTTCGAAGGCACTCCGGCCTACCCATCATTTTCCCGTTACTGGGATATAGTCGATGACCATAAAATAACCCATTTCTATGTGGCGCCTACCGCTCTACGTTTGCTAAAGAGGGCCGGGACTCACCACATCAAACACGACTTGAGCTCACTAAGAACTCTTGGATCTGTTGGCGAACCTATCGCCCCCGACGTATGGCAGTGGTACAATGACAACATAGGCAGAGGAAAAGCACATATCTGTGATACTTACTGGCAAACGGAGACAGGGTCACACATAATTGCGCCCATGGCGGGAGTTACCCCCACAAAGCCCGGCTCCGCCTCATTGCCCGTATTTGGAATTGACCCCGTGATCATTGATCCAGTAAGTGGCGAGGAATTAAAGGGCAATAACGTTGAAGGGGTACTTGCTTTGAGAAGCCCTTGGCCTTCAATGGCCAGAACTGTTTGGAATACGCATGAAAGGTACATGGAAACCTATTTAAGACCCTATCCAGGATATTACTTCACTGGCGACGGGGCTGCGAGGGATAATGACGGATTTTATTGGATCAGGGGCAGAGTTGATGACGTGGTGAATGTGAGTGGTCACAGATTATCAACAGCAGAAATTGAAGCCGCTCTGATTGAACATGCCCAAGTCAGCGAGAGCGCGGTCGTCGGAGTTCATGACGATCTTACAGGTCAAGCGGTGAATGCGTTCGTGGCGCTGAAAAATCCAGTAGAAGACGTCGATGCACTAAGAAAGGAGTTGGTTGTGCAAGTGAGGAAAACAATCGGCCCATTTGCCGCACCTAAAAACGTAATAATTGTGGACGATTTACCCAAAACACGTAGTGGGAAAATTATGAGGAGGATCTTGCGTAAGGTACTAGCCGGTGAGGAGGACCAGTTAGGTGACATCAGCACGCTGGCGAATCCCGACGTCGTACAAACAATCATTGAAGTGGTGCATAGCCTAAAAAAGTAA |
| ACS1_SE_^L641P^ | AAGGAAAACAACTCCATGAGCCAAACTCACAAACACGCGATACCGGCCAACATTGCAGACAGATGCCTAATCAATCCCGAACAATACGAGACAAAGTACAAGCAATCAATCAATGACCCGGACACGTTCTGGGGTGAACAGGGTAAGATTTTAGATTGGATTACGCCGTACCAAAAAGTGAAAAACACCTCATTCGCCCCCGGTAATGTCAGTATTAAGTGGTACGAAGACGGTACCCTGAACTTAGCAGCAAATTGTCTTGACCGTCATCTTCAGGAAAACGGAGATAGAACAGCGATAATCTGGGAGGGCGACGATACCAGTCAAAGTAAGCACATATCCTATAGAGAATTGCACAGGGACGTGTGTAGATTCGCGAATACCTTGCTTGACCTAGGAATTAAAAAAGGCGACGTGGTCGCCATTTACATGCCAATGGTTCCTGAGGCAGCTGTTGCCATGCTTGCGTGTGCACGTATCGGGGCTGTACATAGTGTCATTTTTGGAGGGTTCTCACCTGAGGCGGTGGCAGGTAGAATAATCGACAGTAGTTCCCGTTTGGTGATTACAGCGGATGAAGGTGTCAGGGCAGGTAGGAGCATCCCCCTGAAGAAGAACGTAGACGACGCGCTAAAAAATCCAAACGTTACAAGCGTGGAGCATGTCATTGTTCTGAAAAGAACTGGTTCAGATATTGACTGGCAGGAGGGGCGTGATCTTTGGTGGAGAGATCTGATCGAGAAGGCTTCACCAGAGCATCAGCCTGAGGCCATGAATGCAGAGGACCCCTTATTCATTCTTTATACCAGCGGATCCACGGGTAAGCCAAAAGGGGTTTTGCATACAACTGGTGGTTACCTGGTGTACGCGGCAACGACCTTCAAATACGTCTTCGACTACCATCCGGGAGATATCTACTGGTGTACGGCAGATGTAGGCTGGGTGACAGGCCACTCATACTTACTTTATGGCCCCCTAGCGTGTGGTGCTACCACATTGATGTTCGAGGGTGTGCCAAATTGGCCGACTCCTGCCCGTATGTGCCAGGTGGTTGACAAGCACCAAGTCAACATACTTTATACGGCCCCGACCGCCATTAGGGCATTGATGGCTGAGGGCGACAAGGCAATAGAGGGTACGGATAGATCCTCATTGCGTATCTTGGGTTCCGTGGGAGAACCGATAAATCCAGAAGCGTGGGAATGGTACTGGAAGAAAATAGGCAAAGAAAAGTGCCCCGTGGTTGACACTTGGTGGCAGACAGAAACTGGCGGCTTCATGATCACACCCTTACCAGGGGCCATCGAACTAAAAGCCGGCTCCGCTACTCGTCCCTTCTTCGGTGTACAGCCAGCACTTGTTGATAACGAGGGTCACCCCCAGGAGGGAGCAACGGAGGGGAACTTGGTAATTACGGACTCTTGGCCAGGACAAGCGAGGACTCTATTTGGAGATCACGAGCGTTTTGAACAGACATACTTCAGCACGTTCAAGAACATGTATTTCTCTGGTGACGGAGCAAGGCGTGACGAAGATGGTTACTACTGGATAACTGGTAGAGTTGATGATGTACTTAACGTTTCTGGTCATAGGCTTGGAACTGCCGAAATTGAGTCCGCTTTAGTTGCTCACCCAAAGATTGCGGAAGCCGCAGTCGTAGGCATCCCGCATGCAATAAAAGGACAAGCAATATATGCCTACGTTACACTAAACCACGGCGAAGAGCCATCTCCGGAGCTGTATGCTGAGGTCAGAAATTGGGTGAGGAAGGAGATCGGACCTCTTGCGACCCCCGATGTCCTGCATTGGACGGACAGTCTGCCTAAGACCCGTAGTGGGAAGATCATGAGGCGTATATTGAGGAAGATCGCCGCGGGTGATACTTCAAACCTAGGAGACACTTCTACACTAGCTGACCCTGGTGTAGTGGAGAAGTTGTTGGAGGAGAAGCAAGCCATTGCTATGCCGTCATAA |
| CrtE_Pa_ | ATGGCTTACACAGCTATGGCAGCAGGTACTCAATCCTTGCAGTTGAGAACCGTCGCTTCTTACCAAGAGTGCAACTCCATGAGGTCTTGCTTCAAGTTGACCCCATTCAAGTCCTTCCACGGCGTTAACTTCAACGTCCCATCTTTGGGTGCCGCTAATTGCGAAATCATGGGTCACTTGAAGTTGGGCTCCTTGCCATACAAGCAGTGTTCCGTTTCCTCCAAGTCTACCAAGACTATGGCTCAATTAGTTGATTTGGCAGAAACCGAAAAGGCCGAAGGTAAGGATATCGAGTTCGACTTCAACGAGTACATGAAGAGCAAGGCCGTTGCAGTTGACGCCGCTTTGGATAAGGCTATTCCATTGGAATACCCAGAGAAGATCCACGAGTCCATGAGATACTCTTTGTTGGCCGGAGGTAAGAGAGTCAGACCAGCACTTTGCATTGCCGCTTGCGAATTGGTTGGAGGTTCTCAAGACTTGGCTATGCCAACAGCTTGCGCTATGGAAATGATCCACACCATGTCCTTGATCCACGACGATTTGCCTTGCATGGACAACGACGACTTCAGAAGAGGTAAGCCAACCAACCACAAGGTCTTTGGCGAAGATACAGCCGTTTTGGCAGGTGACGCTTTGTTGTCCTTCGCTTTCGAACACATCGCCGTTGCTACTTCTAAGACCGTTCCATCCGACAGAACCTTGAGAGTCATCTCCGAATTGGGTAAGACCATCGGTAGCCAAGGTTTGGTTGGTGGTCAAGTAGTTGATATTACCTCCGAAGGCGACGCTAACGTTGATTTGAAGACCTTGGAGTGGATTCACATCCATAAGACCGCCGTTCTATTGGAGTGTTCCGTTGTTTCCGGCGGTATTTTGGGAGGAGCTACAGAAGACGAGATTGCCAGAATCAGGAGATACGCTAGGTGCGTTGGTTTGTTGTTCCAGGTGGTTGATGATATCTTGGACGTCACCAAGTCCTCCGAAGAATTGGGTAAGACCGCAGGTAAGGACTTGTTGACCGATAAGGCCACCTACCCAAAGTTGATGGGTTTGGAAAAGGCTAAGGAATTTGCAGCAGAATTGGCTACCAGAGCCAAGGAAGAGTTGTCCTCCTTCGATCAAATCAAAGCCGCTCCATTGTTGGGCTTGGCAGATTACATCGCCTTCAGGCAGAACTGA |
| CrtB_Pag_ | ATGTCCCAACCACCATTGTTGGATCACGCTACCCAAACTATGGCTAACGGTTCTAAGTCCTTCGCTACCGCAGCTAAGTTGTTCGATCCAGCTACTAGGAGGTCCGTCTTGATGTTGTACACTTGGTGCAGACATTGCGACGACGTTATCGACGATCAAACCCACGGTTTCGCTTCAGAAGCAGCTGCCGAAGAAGAAGCTACTCAAAGATTGGCCAGATTGAGGACTTTGACTTTGGCCGCTTTCGAAGGAGCAGAAATGCAAGACCCAGCTTTTGCCGCTTTCCAAGAAGTTGCCTTGACTCACGGTATCACTCCAAGAATGGCCTTGGATCACTTGGACGGTTTTGCTATGGACGTCGCTCAAACCAGATACGTTACCTTCGAGGACACCTTGAGATATTGCTACCACGTTGCAGGCGTTGTGGGTTTGATGATGGCCAGAGTGATGGGTGTCAGAGACGAAAGAGTCTTGGACAGAGCTTGCGATTTGGGTTTGGCTTTCCAGTTGACCAACATCGCTAGGGATATCATCGACGACGCCGCTATTGATAGGTGTTACTTGCCAGCAGAGTGGTTGCAAGACGCAGGTTTAACTCCAGAAAACTACGCCGCCAGAGAAAACAGAGCAGCCTTGGCCAGAGTTGCAGAAAGATTGATCGACGCCGCCGAACCATACTACATTTCTTCTCAAGCCGGTTTGCACGATTTACCACCTAGATGCGCTTGGGCTATTGCTACAGCTAGATCAGTTTACAGGGAGATCGGCATCAAGGTTAAGGCAGCAGGAGGTTCAGCTTGGGATAGAAGACAACACACCTCCAAGGGCGAAAAGATTGCTATGTTGATGGCCGCTCCAGGTCAAGTTATCAGAGCCAAGACCACCAGAGTTACTCCAAGACCAGCAGGTCTTTGGCAAAGACCAGTTTGA |
| CrtI_Bt_ | ATGTCCGACCAGAAGAAGCACATCGTCGTTATTGGCGCAGGTATTGGAGGTACAGCTACTGCTGCTAGATTGGCTAGAGAAGGTTTCAGAGTCACCGTCGTCGAAAAGAACGATTTCTCCGGCGGTAGGTGTTCTTTCATTCATCACGACGGTCATAGATTCGATCAAGGCCCATCCTTGTACTTGATGCCAAAGTTGTTCGAGGACGCCTTCGCAGATTTGGACGAAAGAATCGGCGACCATTTGGACCTATTGAGGTGCGACAACAACTACAAGGTTCATTTCGACGACGGAGACGCAGTTCAGTTGTCCTCCGACTTGACCAAGATGAAGGGCGAATTGGACAGGATTGAAGGTCCATTGGGTTTCGGCAGGTTCTTGGACTTCATGAAGGAGACCCACGTTCACTACGAACAAGGTACTTTTATCGCCATCAAGAGGAACTTCGAGACCATTTGGGACTTGATCAGGTTGCAGTACGTCCCAGAAATTTTTAGGTTGCACTTGTTCGGCAAAATTTATGATAGAGCCTCCAAGTACTTCCAGACCAAGAAGATGAGGATGGCCTTCACCTTCCAGACCATGTACATGGGCATGTCTCCATACGACGCTCCAGCAGTTTACTCTTTGTTGCAGTACACCGAGTTCGCCGAAGGTATTTGGTATCCAAGAGGAGGTTTCAACATGGTCGTCCAGAAGTTGGAATCCATCGCTTCCAAGAAGTACGGCGCAGAATTTAGATATCAATCCCCAGTTGCCAAGATCAACACCGTTGACAAGGACAAGAGAGTTACCGGCGTTACCTTGGAATCCGGTGAAGTTATTGAAGCCGACGCAGTTGTTTGTAACGCCGATTTGGTCTACGCCTACCATCACTTGTTGCCACCTTGTAATTGGACCAAGAAGACCTTGGCCTCTAAGAAGTTGACCTCCTCCTCCATCTCCTTCTATTGGTCCATGTCCACCAAGGTTCCACAATTAGATGTTCACAACATCTTCTTGGCCGAGGCTTACAAGGAGTCCTTCGACGAAATTTTTAACGACTTCGGTTTGCCATCCGAAGCATCTTTCTACGTCAACGTTCCATCCAGAATTGACGAATCCGCAGCTCCACCAAACAAGGACTCCATCATCGTCTTGGTCCCAATCGGTCACATGAAGTCTAAGACCGGTAACTCCGCCGAAGAAAACTACCCAGAATTGGTCAACAGGGCCAGAAAGATGGTGTTGGAAGTCATCGAGAGAAGATTGGGCGTCAACAACTTCGCCAACTTGATCGAGCACGAGGAAGTTAACGATCCATCCGTTTGGCAGTCCAAGTTCAACCTTTGGAGGGGTTCCATCTTGGGTTTGTCTCACGACGTTTTCCAGGTCCTTTGGTTCAGACCATCTACCAAGGACTCCACCAACAGATACGACAACTTGTTCTTCGTCGGCGCTTCTACTCATCCAGGTACAGGCGTTCCAATCGTTTTGGCAGGTTCCAAGTTGACCTCCGATCAGGTTTGCAAGTCCTTCGGTCAAAACCCATTGCCAAGGAAGTTGCAGGACTCTCAGAAGAAGTACGCCCCAGAACAAACCAGAAAGACCGAGTCCCATTGGATCTACTACTGCTTGGCTTGCTACTTCGTCACCTTCTTGTTCTTCTACTTCTTCCCAAGAGACGACACCACTACTCCAGCCTCCTTCATCAACCAGTTGTTGCCAAACGTCTTCCAGGGTCAAAACTCCAACGACATCAGGATCTGA |

**Table S4** Acetate inhibitory gene screened by transcriptome

| **systematic name** | **Gene** | **describe** | **Decline ratio** | |
| --- | --- | --- | --- | --- |
|  |  |  | 2h | 12h |
| YMR251W-A | *HOR7* | Putative protein of unknown function | 0.21 | 0.4 |
| YPL223C | *GRE1* | Hydrophilin essential in desiccation-rehydration process | 0.38 | 0.35 |
| YHR139C | *SPS100* | Protein required for spore wall maturation | 0.22 | 0.46 |
| YLR307C-A | *DPA10* | Putative mitochondrial protein of unknown function | 0.12 | 0.28 |

**Supplementary figures:**


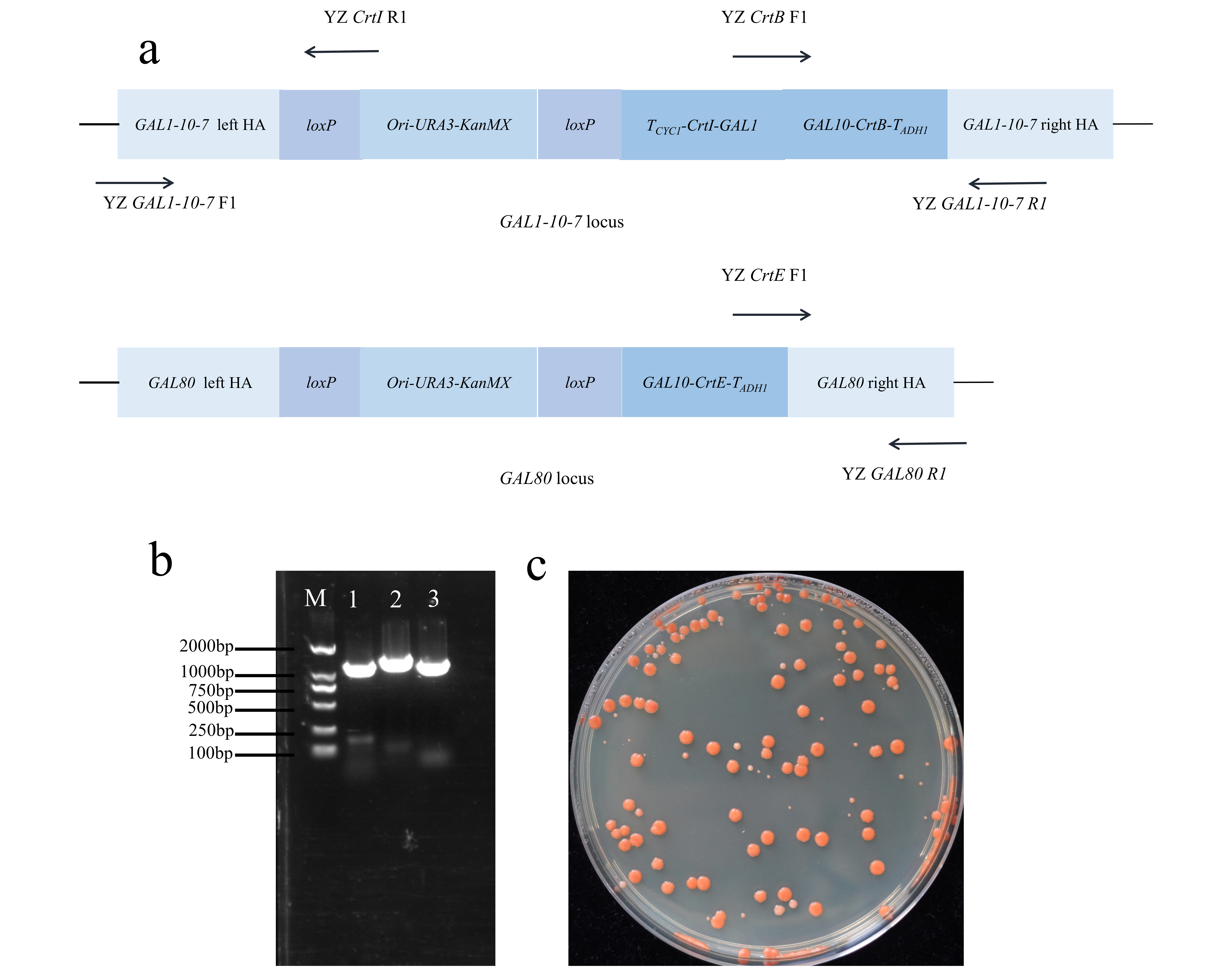


**Figure S1**. PCR verification analysis and plate characterization of strain YLY-01. (**a**) The location of primers for genotype PCR diagnosis of strain YLY-01 (**b**) PCR verification analysis, Note: Lane 1: *CrtE* verification; Lane 2: *CrtB* verification; Lane 3: *CrtI* verification; M: DL2000. (**c**) Plate characterization of strain YLY-01.


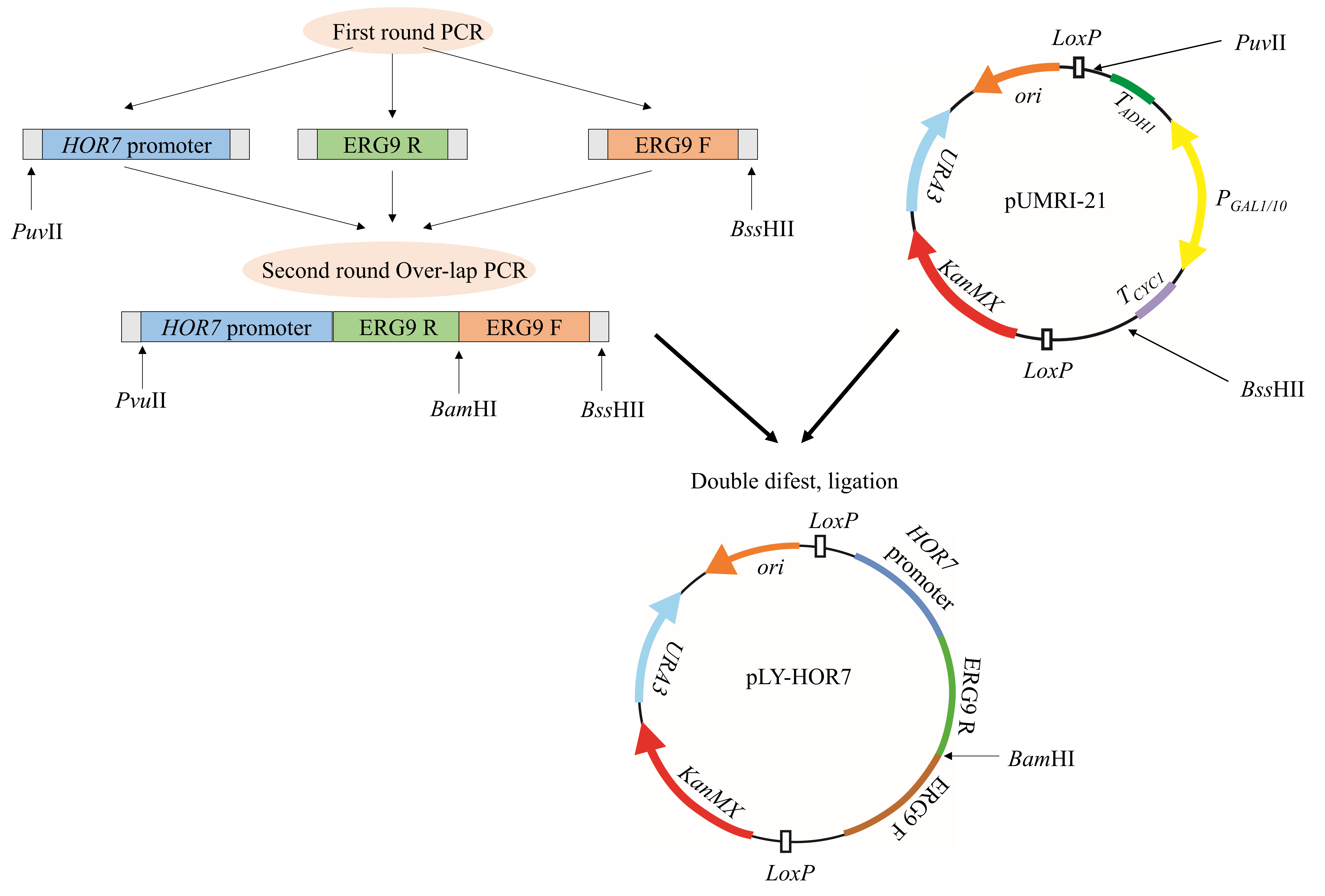


**Figure S2.** Promoter replacement plasmid construction schematic. Take the promoter replacement of *HOR7* gene as an example:

(1) Three fragments of *HOR7* promoter, *ERG9* F (512 bp upstream of *ERG9* promoter) and *ERG9* R (586 bp downstream of *ERG9* promoter) were amplified from genomic DNA of yeast FY1679-01B. The triplex fragment of *HOR7* promoter -*ERG9* R-*ERG9* F was obtained by Overlap extension PCR. The restriction restriction site *Bam*HI was inserted between *ERG9* F and *ERG9* R for subsequent plasmid linearization.

(2) The plasmid pUMRI-2 was double-digested at *Pvu*II and *Bss*HII digestion sites.

(3) The plasmid was connected with the triplet fragment through seamless cloning to obtain the plasmid pLY-*HOR7*.

(4) *Bam*HI linearized plasmid pLY-*HOR7* was used for subsequent yeast transformation.

**
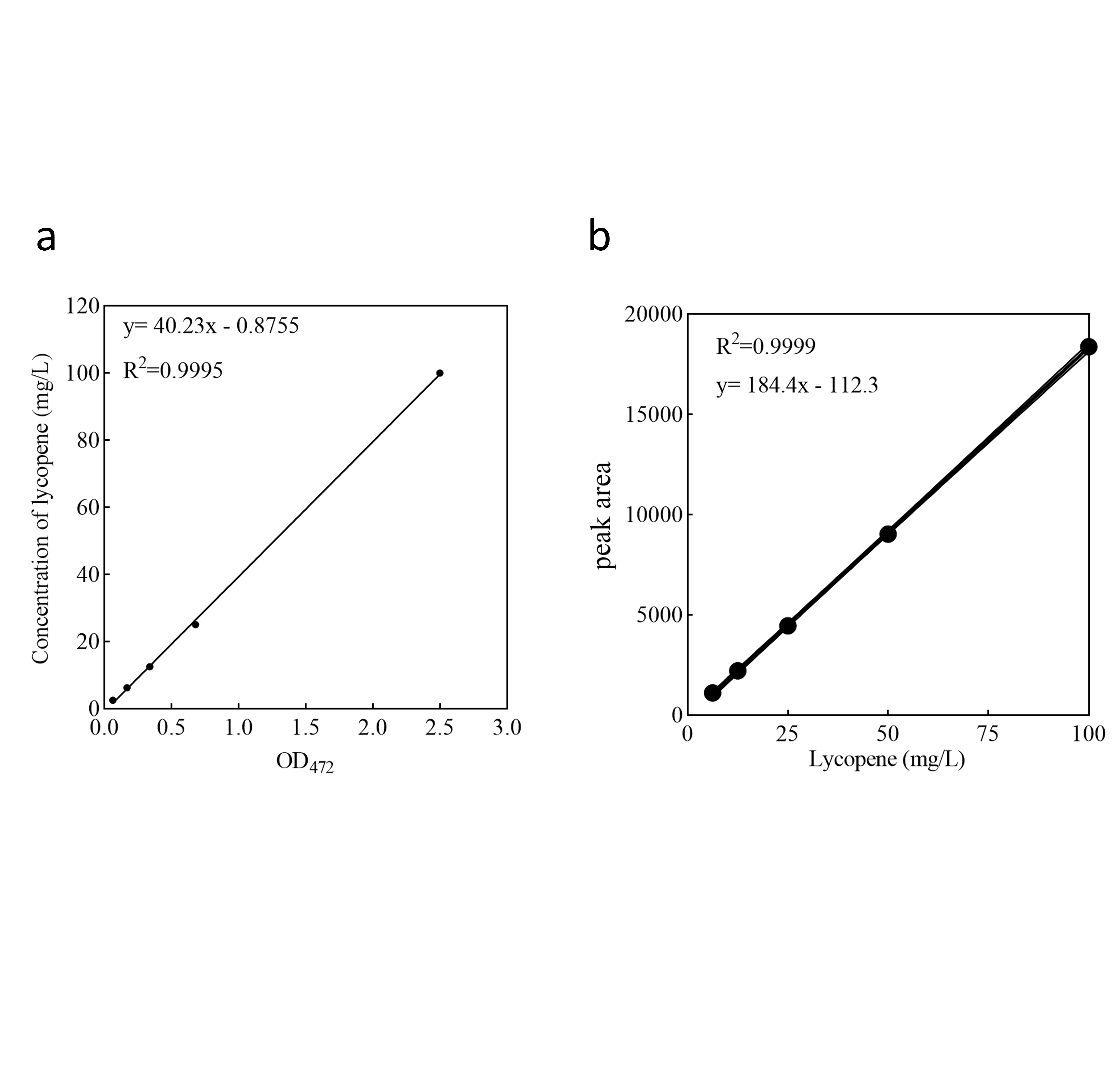
**

**Figure S3. (a)** The standard curve of lycopene. (b) The standard curve of lycopene in dodecane.





**Figure S4.** RT-qPCR validation of the relative expression of target genes in strain YLY-04 at acetate stress 2 h and 12 h.

**
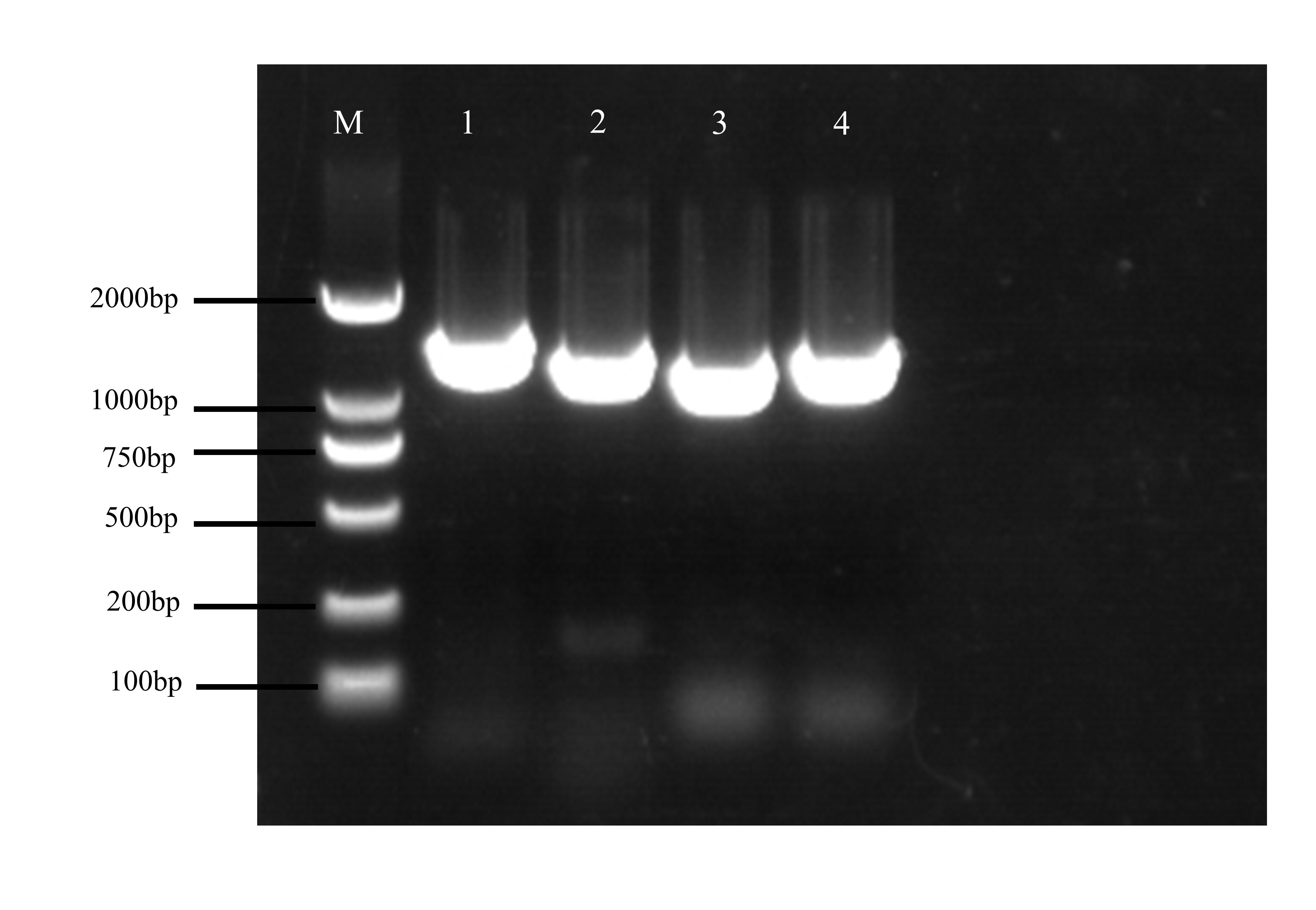
**

**Figure S5.** Genome PCR confirmation of *ERG9* promoter replaced strains. Note: M: Marker DL2000; Lane 1: YLY-46; Lane 2: YLY-47; Lane 3: YLY-48; Lane 4: YLY-49.

**
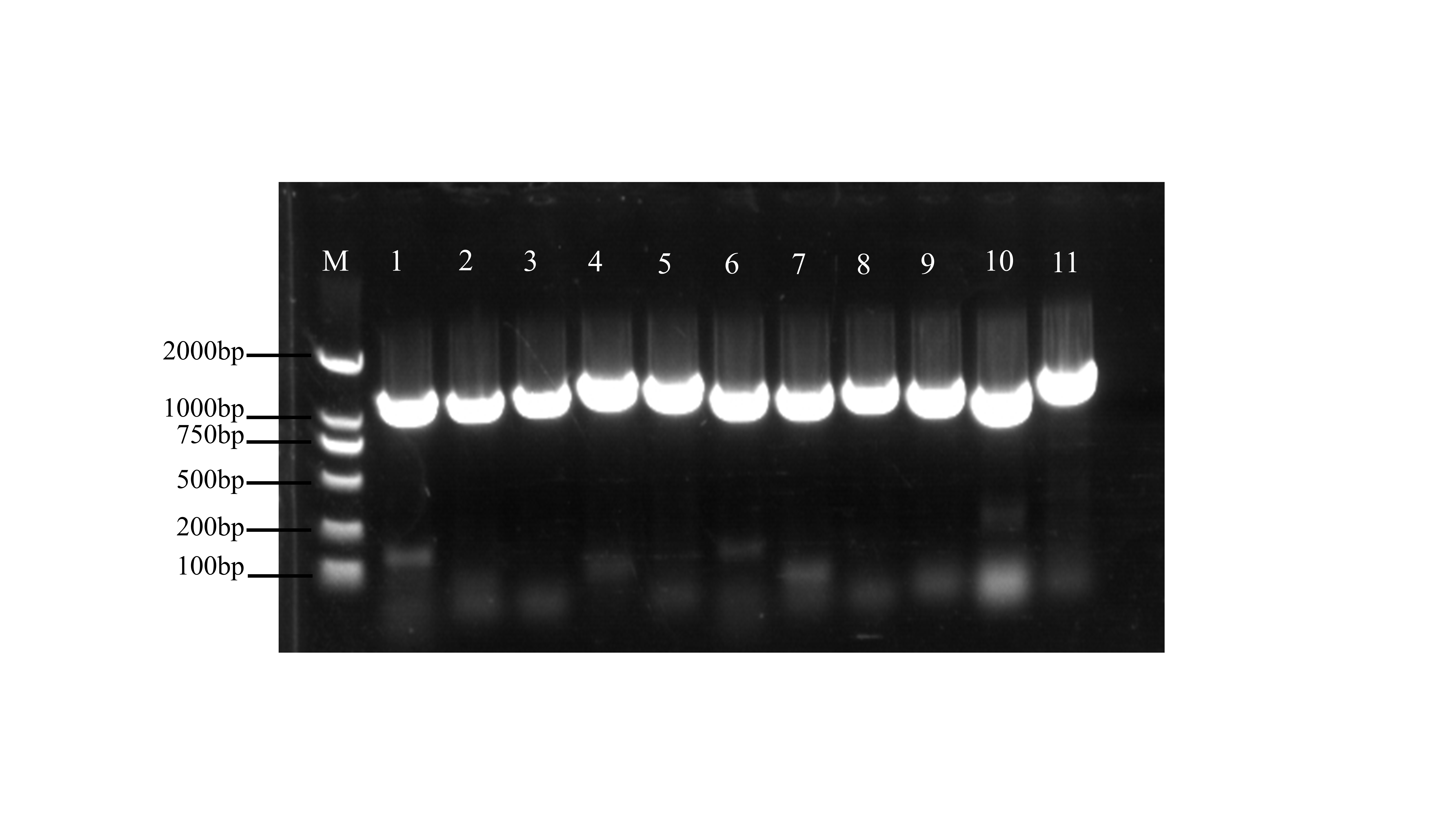
**

**Figure S6**: Genome PCR confirmation of ABC transporter overexpression strains. Note: M: Marker DL2000; Lane 1: YLY-PDR5; Lane 2: YLY-PDR10; Lane 3: YLY-PDR11; Lane 4: YLY-PDR12; Lane 5: YLY-PDR15; Lane 6: YLY-PDR18; Lane 7: YLY-SNQ2; Lane 8: YLY-STE6; Lane 9: YLY-YOR1; Lane 10: YLY -YOL075C; Lane 11: YLY-AUS1.

**References:**

Lv, X., Wang, F., Zhou, P., Ye, L., Xie, W., Xu, H., & Yu, H. (2016). Dual regulation of cytoplasmic and mitochondrial acetyl-CoA utilization for improved isoprene production in *Saccharomyces cerevisiae*. *Nature Communications*, *7*(1), Article 1. https://doi.org/10.1038/ncomms12851
